# Commonalities in biomarkers and phenotypes between mild cognitive impairment and cerebral palsy: a pilot exploratory study

**DOI:** 10.18632/aging.202563

**Published:** 2021-01-26

**Authors:** Ted Kheng Siang Ng, Alex Tagawa, Roger Chun-Man Ho, Anis Larbi, Ee Heok Kua, Rathi Mahendran, James J. Carollo, Patricia C. Heyn

**Affiliations:** 1 Department of Psychological Medicine, Yong Loo Lin School of Medicine, National University of Singapore, Singapore, Singapore; 2 Children’s Hospital Colorado, Center for Gait and Movement Analysis (CGMA), Aurora, CO 80045, USA; 3 Department of Psychological Medicine, National University Hospital, Singapore, Singapore; 4 Biomedical Global Institute of Healthcare Research and Technology (BIGHEART), National University of Singapore, Singapore, Singapore; 5 Center of Excellence in Behavioral Medicine, Nguyen Tat Thanh University, Ho Chi Minh City, Vietnam; 6 Faculty of Education, Huaibei Normal University, Huaibei, China; 7 Singapore Immunology Network, Agency for Science, Technology and Research, Singapore, Singapore; 8 University of Colorado Anschutz Medical Campus, Aurora, CO 80045, USA; 9 Academic Development Department, Duke-NUS Medical School, Singapore, Singapore

**Keywords:** biomarker, aging model, comparative study, community-dwelling older adults, cerebral palsy

## Abstract

Clinically, individuals with cerebral palsy (CP) experience symptoms of accelerated biological aging. Accumulative deficits in both molecular underpinnings and functions in young adults with CP can lead to premature aging, such as heart disease and mild cognitive impairment (MCI). MCI is an intermediate stage between healthy aging and dementia that normally develops at old age. Owing to their intriguingly parallel yet “inverted” disease trajectories, CP might share similar pathology and phenotypes with MCI, conferring increased risk for developing dementia at a much younger age. Thus, we examined this hypothesis by evaluating these two distinct populations (MCI= 55, CP = 72). A total of nine measures (e.g., blood biomarkers, neurocognition, Framingham Heart Study Score (FHSS) were compared between the groups. Compared to MCI, upon controlling for covariates, delta FHSS, brain-derived neurotrophic factor (BDNF) levels, and systolic blood pressure were significantly lower in CP. Intriguingly, high-sensitivity CRP, several metabolic outcomes, and neurocognitive function were similar between the two groups. This study supports a shared biological underpinning and key phenotypes between CP and MCI. Thus, we proposed a double-hit model for the development of premature aging outcomes in CP through shared biomarkers. Future longitudinal follow-up studies are warranted to examine accelerated biological aging.

## INTRODUCTION

Dementia is an overall term for diseases, conditions, and syndromes that are characterized by a decline in cognition that affects a person’s daily functional ability [1]. In aging populations, dementia has multiple etiologies, including infections, low-grade systemic inflammation, metabolic and cardiovascular dysregulations, resulting in neurodegeneration and subsequent manifestations of clinical symptoms. While dementia is typically associated and diagnosed in the geriatric population, it is not uncommon to see signs of dementia in the younger population. Current literature reports that approximately 67-98 per 100,000 people aged 45-64 years old have early-onset dementia, in which these individuals experience significant pathology, behavioral changes, psychiatric manifestation, and cognitive decline [1, 2]. Additionally, many neurodegenerative disorders can cause early-onset dementia, for example Behcet’s disease, in which the onset can be observed in individuals as young as 20 years old [1]. Alzheimer’s Disease (AD) has also been well-established in Down syndrome (DS) [3, 4], although not everyone with DS develops AD symptoms, autopsy studies have shown that by age of 40 years old, the brains of almost all individuals with DS have significant levels of beta-amyloid plaques and tau tangles, abnormal protein deposits considered AD hallmarks [5].

To detect early dementia, many clinicians utilize screening tools to look for signs of mild cognitive impairment (MCI). MCI is defined as the clinical stage between the expected cognitive decline of normal healthy aging and a more serious decline characterizing dementia. In the literature, the conversion from MCI to dementia is associated with increased inflammation and decreased neurotrophic factors [6]. While studies on dementia and MCI focus mostly on either the geriatric population or older adults with neurodegenerative disorders and conditions, the current literature has yet to address early screening and risk factors identification in younger populations who might be at higher risk for cognitive impairment, due to lifelong co-existing functional deficits and health risk factors accumulation, such as individuals with developmental disabilities [7–9]. Thus, these young adults may have high-risk of developing premature or accelerated aging-related diseases. Specifically, adults with cerebral palsy (CP), who usually presents with both physical and cognitive impairments, need to be included in studies evaluating risk factors for dementia [10, 11].

CP is characterized by damages or malformations of the brain sustained before, during, or shortly after birth and it affects 2 – 2.5 individuals out of 1000 live births, making it the most common physical disability in children [12, 13]. Although CP is considered a childhood condition, it is a chronic disability that presents challenges throughout one’s lifetime [14–16]. Recently, there have been reports that transitioning to adulthood, adults with CP are at greater risk for developing secondary health conditions that could be the clinical manifestations of accelerated aging, such as cardiovascular diseases [14–16]. Due to the risk of accelerated aging and impairments in various physiological systems, adults with CP may prematurely develop cognitive impairments at a young age, similar to how older adults develop MCI. Evidence in the literature support the notion that the damages in the brain experienced by individuals with CP could cause persistent inflammation and immune dysfunction [17, 18]. This immune dysfunction and heightened secretion of inflammatory byproducts are similar to the heightened immune activities observed in older adults with MCI, such as increased secretion of cytokines and low-grade systemic inflammation [19]. Furthermore, although the BDNF levels in MCI have been inconclusive, its level is usually significantly decreased in patients with AD [20]. To our knowledge, an examination of BDNF levels in adults with CP has not been performed previously either.

In CP, cognitive impairments often manifest in adulthood, after years of having physical impairments sustained since childhood. Whereas in MCI, cognitive impairments typically precede physical impairments. Owing to their intriguingly parallel yet “inverted” disease trajectories, CP and MCI might share multiple similar biological underpinnings and phenotypes. However, to our best knowledge, there is currently no literature comparing the two populations in a single study. Specifically, investigation on which are the commonalities between CP and MCI could improve our understanding of the risk and the pathophysiology of developing dementia in adults with CP, thus informing preventive measures. Hence, we conducted this post-hoc exploratory study to address this gap in knowledge. As such, this study has three aims. Aim 1 investigated if there are both common and distinct biomarkers and phenotypes between adults with CP and MCI. Aim 2 examined if the biomarkers were significantly associated with the phenotypes. Aim 3 examined if the associations between measures were largely attributed to the effects of aging or pathophysiology.

## RESULTS

### Cohorts baseline characteristics

Table 1A summarizes the baseline characteristics of the study participants. We recruited a total of 127 participants, aged mean=24.97, SD=5.29 (CP cohort) and mean=71.28, SD=6.03 (MCI cohort). Most of the participants were female in the MCI cohort (70.8%), while the CP cohort had a balanced number of genders, with 48.1% female (Table 1A, 1B). Years of formal education also differed significantly between CP and MCI cohorts (mean, SD, CP=13.49±2.25 years, MCI=4.31±4.66 years). No significant differences in BMI were observed, but all the other eight outcome variables were significantly different based on bivariate testing. Table 1B presented the specific clinical characteristics of the study participants for both diagnostic entities, specifically the CP diagnoses or subtypes, i.e. quadriplegic, hemiplegic, diplegic, or triplegic. Furthermore, we also presented the distributions of the GMFCS levels of CP and the two MCI subtypes for patients with MCI.

**Table 1A:** Demographics characteristics of study participants.

**Cohorts**	**CP (n=72)**	**MCI (n=55)**	***P*-values**
**Demographics Characteristics (Total N=127)**	**mean ± SD *or* n (%)**	**mean ± SD *or* n (%)**	
Age (in years)	24.97±5.29	71.28±6.03	<0.001***
Gender			
Female	38 (48.1%)	34 (70.8%)	0.01*
Male	41 (51.9%)	14 (29.2%)	
Years of Formal Education	13.49±2.25	4.31±4.66	<0.001***
Log-transformed hs-CRP	-0.05±0.58	0.21±0.44	0.014*
Log-transformed BDNF	2.07±0.52	7.34±0.85	<0.001***
Semantic Fluency (60-second animal naming)	16.93±6.07	12.56±2.98	<0.001***
WAIS-V Block Design	26.97±14.94	19.09±9.15	0.001**
BMI	24.22±5.51	24.42±3.84	0.815
Resting heart rate (bpm)	79.44±12.37	70.93±9.99	<0.001***
Systolic blood pressure	122.03±12.93	138.31±21.09	<0.001***
Diastolic blood pressure	76.59±7.59	72.45±10.81	0.019*
Natural log-transformed delta FHS score	-0.02±0.34	2.26±0.91	<0.001***

Footnotes: CP= cerebral palsy; MCI=mild cognitive impairment; hs-CRP=high-sensitivity C-reactive protein; BDNF=brain-derived neurotrophic factor; WAIS= Wechsler Adult Intelligence Scale, BMI= Body-mass index; bpm= beats per minute; FHS=Framingham heart study; * indicates <0.05, ** indicates <0.01, *** indicates <0.001.

**Table 1B:** Clinical characteristics of study participants, specific to diagnostic entities.

**CP (n=72)**	
**CP Diagnoses/ Subtypes, N (%)**	
Hemiplegic	27 (37.5%)
Diplegic	38 (52.7%)
Triplegic	4 (5.6%)
Quadraplegic	3 (4.2%)
**STMS**	
Screened as normal cognition	18 (25.7%)
Screened as MCI	52 (74.3%)
**GMFCS, N (%)**	
I	28 (38.9%)
II	29 (40.3%)
III	13 (18.1%)
IV	2 (2.7%)
V	0 (0%)
**MCI (n=55)**	
**MCI Subtypes**	
Amnestic MCI	21 (38.2%)
Non-amnestic MCI	34 (61.8%)

Footnotes: STMS= Short Test of Mental Status; GMFCS=Gross Motor Function Classification System; the total n for STMS was only 70, due to two of the participants not having complete data for this measure; STMS cut-off for MCI was 34.5.

### Commonalities between the CP and MCI cohorts through overlapping biomarkers and phenotypes

Based on the results from Table 2, there were six biomarkers and phenotypes that were statistically non-significantly different between CP and MCI. The results remained statistically insignificant upon controlling for all the available covariates in their respective models 3. They were hs-CRP levels (model 3: β= 0.221, 95% CI=-0.074 to 0.515, p=0.141), semantic fluency test (model 3: β= -1.933, 95% CI=-4.713 to 0.847, p=0.171), WAIS-V Block Design (model 3: β= 2.834, 95% CI=-3.989 to 9.657, p=0.412), BMI (model 3: β= -0.538, 95% CI=-3.334 to 2.258, p=0.704), resting heart rate (model 3: β= -4.583, 95% CI=-10.914 to 1.748, p=0.154), and diastolic BP (model 3: β= -0.226, 95% CI=-5.376 to 4.924, p=0.931).

**Table 2:** Shared and distinct biomarkers, neurocognitive, and anthropometric measures between adults with CP and MCI.

**Dependent variable: Biomarkers /Neurocognitive /Anthropometric measures**	**Models**	**Independent variable: CP versus MCI cohorts**				
		**β (95% CI)**	***P*-value of regression models**	***R^2^***	***R^2^* Change**	***P*-value of *R^2^* Change**
Log-transformed hs-CRP	1	0.218(0.04-0.397)	0.017*	0.045	0.045	0.017*
	2	0.215(0.031-0.399)	0.022*	0.045	0	0.88
	3	0.221(-0.074-0.515)	0.141	0.045	0	0.964
Log-transformed BDNF	1	4.155(3.719-4.59)	<0.001***	0.74	0.74	<0.001***
	2	4.176(3.728-4.624)	<0.001***	0.741	0	0.675
	3	3.976(3.26-4.693)	<0.001***	0.742	0.001	0.481
Semantic Fluency#	1	-4.225(-5.941--2.51)	<0.001***	0.16	0.16	<0.001***
	2	-4.248(-6.014--2.481)	<0.001***	0.16	0	0.911
	3	-1.933(-4.713-0.847)	0.171	0.189	0.029	0.036*
WAIS-V Block Design	1	-7.58(-11.96--3.201)	0.001**	0.086	0.086	0.001**
	2	-7.551(-12.061--3.04)	0.001**	0.086	0	0.954
	3	2.834(-3.989-9.657)	0.412	0.185	0.099	<0.001***
BMI (kg/m^2^)	1	0.189(-1.517-1.895)	0.827	0	0	0.827
	2	-0.054(-1.8-1.693)	0.951	0.013	0.012	0.218
	3	-0.538(-3.334-2.258)	0.704	0.014	0.002	0.661
Resting heart rate (bpm)	1	-7.944(-11.816--4.072)	<0.001***	0.117	0.117	<0.001***
	2	-8.143(-12.128--4.158)	<0.001***	0.118	0.001	0.657
	3	-4.583(-10.914-1.748)	0.154	0.132	0.014	0.156
Systolic blood pressure	1	15.295(9.402-21.188)	<0.001***	0.174	0.174	<0.001***
	2	16.235(10.212-22.258)	<0.001***	0.187	0.013	0.167
	3	21.939(12.38-31.499)	<0.001***	0.202	0.015	0.132
Diastolic blood pressure	1	-3.927(-7.092--0.762)	0.015*	0.046	0.046	0.015*
	2	-3.647(-6.899--0.396)	0.028*	0.05	0.004	0.446
	3	-0.226(-5.376-4.924)	0.931	0.072	0.021	0.094
Natural log-transformed delta FHS score	1	1.704(1.446-1.963)	<0.001***	0.577	0.577	<0.001***
	2	1.781(1.523-2.04)	<0.001***	0.6	0.023	0.009**
	3	2.017(1.606-2.428)	<0.001***	0.606	0.007	0.149

Footnotes: CP= cerebral palsy; MCI=mild cognitive impairment; hs-CRP=high-sensitivity C-reactive protein; BDNF=brain-derived neurotrophic factor; #Semantic fluency (60-second animal naming); WAIS= Wechsler Adult Intelligence Scale; BMI= Body-mass index; bpm= beats per minute; FHS=Framingham heart study; 95% CI=95% confidence interval. * indicates <0.05, ** indicates <0.01, *** indicates <0.001. Model 1: no covariates, Model 2: added gender, Model 3: added years of formal education (in years).

### Distinct biomarkers, neurocognitive, and anthropometric measures between CP and MCI cohorts

The natural log-transformed delta FHS score was significantly different between the two cohorts (model 3: β= 2.017, 95% CI=1.606 to 2.428, p<0.001), with CP participants having significantly lower natural log-transformed delta FHS score compared to MCI cohort, after controlling for covariates (Table 2). Similarly, a significant difference in plasma log-transformed BDNF levels between CP and MCI was observed (model 3: β= 3.976, 95% CI=3.26 to 4.693, p<0.001), with CP participants having significantly lower log-transformed BDNF levels compared to MCI cohort, after controlling for covariates (Table 2). After controlling for covariates, systolic blood pressure was also higher in MCI (model 3: β= 21.939, 95% CI=12.38 to 31.499, p<0.001). Without controlling for any covariates, the other measures, except BMI, had significant differences. Nonetheless, after controlling for covariates, the other six measures had no significant differences between CP and MCI cohorts.

### Associations of hs-CRP with biomarkers, neurocognitive, and anthropometric measures

Log-transformed hs-CRP was significantly associated with log-transformed BDNF (model 1: β=1.255, 95% CI=0.458 to 2.052, p=0.002). The relationship became borderline significant after adjusting for additional covariates in the final model (model 4: β=0.425, 95% CI= -0.004 to 0.854, p=0.052) (Table 3). Furthermore, log-transformed hs-CRP was also significantly associated with BMI (model 1: β= 3.032, 95% CI= 1.469 to 4.595), p<0.001 and model 4: β= 3.122, 95% CI=1.516 to 4.727, p<0.001). Lastly, log-transformed hs-CRP was also significantly associated with natural log-transformed delta FHS score (model 4: β= 0.266, 95% CI=0.021 to 0.511, p=0.034).

**Table 3:** Associations of hs-CRP with biomarker, neurocognitive, and anthropometric measures.

**Dependent variable: Biomarkers /Neurocognitive /Anthropometric measures**	**Models**	**Independent variable: Log-transformed hs-CRP**				
		**β (95% CI)**	***P*-value of regression models**	***R^2^***	***R^2^* Change**	***P*-value of *R^2^* Change**
Log-transformed BDNF	1	1.255(0.458-2.052)	0.002**	0.072	0.072	0.002**
	2	1.211(0.42-2.002)	0.003**	0.097	0.025	0.068
	3	0.734(0.143-1.325)	0.015*	0.512	0.416	<0.001***
	4	0.425(-0.004-0.854)	0.052	0.75	0.237	<0.001***
Semantic Fluency#	1	-1.223(-3.022-0.576)	0.181	0.014	0.014	0.181
	2	-1.178(-2.983-0.627)	0.199	0.02	0.005	0.414
	3	-0.53(-2.209-1.149)	0.533	0.179	0.16	<0.001***
	4	-0.383(-2.072-1.306)	0.654	0.191	0.011	0.196
WAIS-V Block Design	1	-1.938(-6.36-2.484)	0.387	0.006	0.006	0.387
	2	-1.839(-6.278-2.6)	0.414	0.01	0.004	0.465
	3	-0.202(-4.309-3.904)	0.922	0.181	0.17	<0.001***
	4	-0.435(-4.583-3.713)	0.836	0.185	0.005	0.403
BMI (kg/m^2^)	1	3.032(1.469-4.595)	<0.001***	0.106	0.106	<0.001***
	2	2.98(1.416-4.545)	<0.001***	0.114	0.009	0.274
	3	3.024(1.434-4.614)	<0.001***	0.115	0.001	0.727
	4	3.122(1.516-4.727)	<0.001***	0.121	0.006	0.366
Resting heart rate (bpm)	1	-4.259(-8.177--0.342)	0.033*	0.036	0.036	0.033*
	2	-4.221(-8.16--0.282)	0.036*	0.037	0.001	0.751
	3	-3.095(-6.886-0.696)	0.109	0.136	0.1	<0.001***
	4	-2.78(-6.597-1.038)	0.152	0.147	0.011	0.219
Systolic blood pressure	1	5.449(-0.756-11.655)	0.085	0.024	0.024	0.085
	2	5.505(-0.735-11.745)	0.083	0.024	0.001	0.77
	3	4.163(-1.992-10.319)	0.183	0.081	0.057	0.007**
	4	2.463(-3.332-8.259)	0.402	0.206	0.125	<0.001***
Diastolic blood pressure	1	-0.526(-3.663-2.61)	0.74	0.001	0.001	0.74
	2	-0.408(-3.543-2.727)	0.797	0.013	0.012	0.214
	3	0.273(-2.818-3.364)	0.861	0.072	0.059	0.006**
	4	0.296(-2.835-3.427)	0.852	0.072	0	0.912
Natural log-transformed delta FHS score	1	0.6(0.23-0.969)	0.002**	0.076	0.076	0.002**
	2	0.599(0.227-0.971)	0.002**	0.076	0	0.944
	3	0.422(0.102-0.741)	0.01*	0.341	0.265	<0.001***
	4	0.266(0.021-0.511)	0.034*	0.621	0.279	<0.001***

Footnotes: CP= cerebral palsy; MCI=mild cognitive impairment; hs-CRP=high-sensitivity C-reactive protein; BDNF=brain-derived neurotrophic factor; #Semantic fluency (60-second animal naming); WAIS= Wechsler Adult Intelligence Scale, BMI= Body-mass index; bpm= beats per minute; FHS=Framingham heart study; 95% CI=95% confidence interval. * indicates <0.05, ** indicates <0.01, *** indicates <0.00. Model 1: no covariates, Model 2: added gender, Model 3: added years of formal education (in years), Model 4: added “CP VS MCI cohort”.

### Associations of natural log-transformed delta FHS score with biomarker, neurocognitive, and anthropometric measures

In Table 4A, we showed that in the CP cohort, the natural log-transformed delta FHS score was significantly associated with log-transformed hs-CRP. The relationship was significant after adjusting for additional covariates in the final model (model 4: β=0.509, 95% CI= 0.044 to 0.974, p=0.032). Natural log-transformed delta FHS score was also significantly associated with semantic fluency through adjusted models (model 3: β=5.705, 95% CI=0.997 to 10.413, p=0.018). Chronological age was a not significant covariate for all the variables of which natural log-transformed delta FHS score regressed on, except with long-transformed hs-CRP, with the addition of chronological age, the model was significantly improved (R^2^ change=0.071, p-value of R^2^ =0.025). Whereas in the MCI cohort, Table 4B, natural log-transformed delta FHS score had two significant associations upon adjusting for covariates, namely with BMI and systolic blood pressure.

**Table 4A:** Associations of natural log-transformed delta FHS score with biomarker, neurocognitive, and anthropometric measures for CP cohort.

**Dependent variable: Biomarkers /Neurocognitive /Anthropometric measures**	**Models**	**Independent variable: Natural log-transformed delta FHS score**				
		**β (95% CI)**	***P*-value of regression models**	***R^2^***	***R^2^* Change**	***P*-value of *R^2^* Change**
Log-transformed hs-CRP	1	0.224(-0.183-0.632)	0.276	0.017	0.017	0.276
	2	0.274(-0.15-0.698)	0.202	0.028	0.011	0.386
	3	0.284(-0.151-0.718)	0.197	0.028	0.001	0.813
	4	0.509(0.044-0.974)	0.032*	0.099	0.071	0.025*
Log-transformed BDNF	1	0.059(-0.288-0.406)	0.734	0.002	0.002	0.734
	2	0.155(-0.197-0.507)	0.383	0.058	0.056	0.046*
	3	0.131(-0.229-0.491)	0.469	0.065	0.007	0.487
	4	0.152(-0.248-0.553)	0.45	0.066	0.001	0.803
Semantic Fluency#	1	5.11(1.118-9.102)	0.013*	0.085	0.085	0.013*
	2	5.57(1.417-9.722)	0.009**	0.094	0.009	0.411
	3	5.26(1.02-9.501)	0.016*	0.102	0.008	0.441
	4	5.705(0.997-10.413)	0.018*	0.105	0.003	0.657
WAIS-V Block Design	1	-1.344(-11.533-8.845)	0.793	0.001	0.001	0.793
	2	0.386(-10.151-10.922)	0.942	0.022	0.021	0.224
	3	-1.861(-12.274-8.553)	0.723	0.093	0.07	0.025*
	4	-2.246(-13.821-9.329)	0.7	0.093	0	0.876
BMI (kg/m^2^)	1	0.146(-3.787-4.079)	0.941	0	0	0.941
	2	0.707(-3.373-4.787)	0.731	0.015	0.015	0.307
	3	0.574(-3.607-4.756)	0.785	0.017	0.002	0.736
	4	0.303(-4.343-4.95)	0.897	0.018	0.001	0.784
Resting heart rate (bpm)	1	-0.655(-8.898-7.587)	0.874	0	0	0.874
	2	1.265(-7.176-9.706)	0.766	0.041	0.04	0.093
	3	0.965(-7.685-9.615)	0.825	0.043	0.002	0.713
	4	0.877(-8.74-10.494)	0.856	0.043	0	0.966
Systolic blood pressure	1	2.506(-6.158-11.17)	0.566	0.005	0.005	0.566
	2	2.648(-6.408-11.704)	0.562	0.005	0	0.907
	3	2.779(-6.509-12.067)	0.553	0.005	0	0.882
	4	1.575(-8.728-11.878)	0.761	0.01	0.005	0.583
Diastolic blood pressure	1	4.621(-0.362-9.604)	0.069	0.047	0.047	0.069
	2	4.994(-0.204-10.192)	0.059	0.051	0.004	0.593
	3	5.261(-0.06-10.581)	0.053	0.055	0.004	0.596
	4	5.42(-0.495-11.335)	0.072	0.055	0	0.899

Footnotes: CP= cerebral palsy; MCI=mild cognitive impairment; hs-CRP=high-sensitivity C-reactive protein; BDNF=brain-derived neurotrophic factor; #Semantic fluency (60-second animal naming); WAIS= Wechsler Adult Intelligence Scale, BMI= Body-mass index; bpm= beats per minute; FHS=Framingham heart study; 95% CI=95% confidence interval. * indicates <0.05, ** indicates <0.01, *** indicates <0.001. Model 1: no covariates, Model 2: added gender, Model 3: added years of formal education (in years), Model 4: added chronological age (in years).

**Table 4B:** Associations of natural log-transformed delta FHS score with biomarker, neurocognitive, and anthropometric measures for MCI cohort.

**Dependent variable: Biomarkers /Neurocognitive /Anthropometric measures**	**Models**	**Independent variable: Natural log-transformed delta FHS score**				
		**β (95% CI)**	***P*-value of regression models**	***R^2^***	***R^2^* Change**	***P*-value of *R^2^* Change**
Log-transformed hs-CRP	1	0.073(-0.073-0.218)	0.321	0.019	0.019	0.321
	2	0.069(-0.083-0.22)	0.368	0.019	0.001	0.842
	3	0.07(-0.084-0.223)	0.367	0.02	0.001	0.851
	4	0.071(-0.086-0.227)	0.368	0.02	0	0.91
Log-transformed BDNF	1	0.193(-0.079-0.465)	0.161	0.037	0.037	0.161
	2	0.236(-0.045-0.516)	0.098	0.063	0.026	0.237
	3	0.244(-0.036-0.524)	0.086	0.084	0.021	0.281
	4	0.262(-0.022-0.546)	0.07	0.099	0.015	0.365
Semantic Fluency#	1	-0.795(-1.914-0.324)	0.16	0.037	0.037	0.16
	2	-0.933(-2.093-0.227)	0.113	0.053	0.016	0.356
	3	-0.998(-2.126-0.129)	0.082	0.125	0.073	0.045*
	4	-0.908(-2.043-0.227)	0.115	0.148	0.023	0.251
WAIS-V Block Design	1	0.974(-2.515-4.464)	0.578	0.006	0.006	0.578
	2	-0.367(-3.711-2.978)	0.827	0.164	0.158	0.003**
	3	-0.617(-3.761-2.526)	0.695	0.279	0.114	0.006**
	4	-0.137(-3.187-2.913)	0.929	0.347	0.069	0.026*
BMI (kg/m^2^)	1	1.13(-0.251-2.511)	0.107	0.048	0.048	0.107
	2	1.354(-0.069-2.776)	0.062	0.075	0.027	0.225
	3	1.407(-0.008-2.822)	0.051	0.107	0.032	0.184
	4	1.546(0.132-2.96)	0.033*	0.142	0.035	0.158
Resting heart rate (bpm)	1	-1.047(-4.826-2.731)	0.581	0.006	0.006	0.581
	2	-2.086(-5.87-1.698)	0.274	0.087	0.081	0.037*
	3	-2.139(-5.959-1.68)	0.266	0.091	0.004	0.62
	4	-1.833(-5.678-2.012)	0.343	0.115	0.024	0.251
Systolic blood pressure	1	12.413(5.187-19.639)	0.001**	0.183	0.183	0.001**
	2	11.414(3.941-18.886)	0.003**	0.2	0.017	0.3
	3	11.202(3.715-18.69)	0.004**	0.215	0.016	0.319
	4	10.77(3.184-18.356)	0.006**	0.226	0.011	0.41
Diastolic blood pressure	1	-0.11(-4.246-4.026)	0.958	0	0	0.958
	2	-0.722(-4.993-3.549)	0.736	0.024	0.024	0.267
	3	-0.846(-5.123-3.432)	0.693	0.043	0.02	0.309
	4	-0.13(-4.236-3.977)	0.95	0.153	0.109	0.014*

Footnotes: CP= cerebral palsy; MCI=mild cognitive impairment; hs-CRP=high-sensitivity C-reactive protein; BDNF=brain-derived neurotrophic factor; #Semantic fluency (60-second animal naming); WAIS= Wechsler Adult Intelligence Scale, BMI= Body-mass index; bpm= beats per minute; FHS=Framingham heart study; 95% CI=95% confidence interval. * indicates <0.05, ** indicates <0.01, *** indicates <0.001. Model 1: no covariates, Model 2: added gender, Model 3: added years of formal education (in years), Model 4: added chronological age (in years).

### Associations of BDNF with biomarkers, neurocognitive, and anthropometric measures

In the CP cohort, log-transformed BDNF was significantly associated with log-transformed hs-CRP (model 1: β=0.355, 95% CI= 0.085 to 0.624, p=0.011) (Table 5A). The relationship remained significant after adjusting for additional covariates through the final model (model 4: β=0.36, 95% CI=0.082 to 0.639, p=0.012). Log-transformed BDNF was also significantly associated with BMI through adjusted models (model 1: β= 2.998, 95% CI= 0.393 to 5.602, p=0.025 and model 4: β= 2.814, 95% CI=0.079 to 5.550, p=0.044). Chronological age was not significant covariate for all the variables of which log-transformed BDNF regressed on Table 5A, except for the natural log-transformed delta FHS score.

**Table 5A:** Associations of BDNF with biomarker, neurocognitive, and anthropometric measures CP cohort.

**Dependent variable: Biomarkers /Neurocognitive /Anthropometric measures**	**Models**	**Independent variable: Log-transformed BDNF**				
		**β (95% CI)**	***P*-value of regression models**	***R^2^***	***R^2^* Change**	***P*-value of *R^2^* Change**
Log-transformed hs-CRP	1	0.355(0.085-0.624)	0.011*	0.09	0.09	0.011*
	2	0.355(0.077-0.633)	0.013*	0.09	0	1
	3	0.358(0.077-0.64)	0.013*	0.091	0.001	0.802
	4	0.36(0.082-0.639)	0.012*	0.122	0.032	0.125
Semantic Fluency#	1	0.205(-2.661-3.071)	0.887	0	0	0.887
	2	0.178(-2.78-3.136)	0.905	0	0	0.933
	3	-0.004(-2.969-2.96)	0.998	0.021	0.021	0.233
	4	-0.013(-2.993-2.966)	0.993	0.027	0.005	0.545
WAIS-V Block Design	1	3.627(-3.319-10.574)	0.301	0.015	0.015	0.301
	2	2.806(-4.307-9.919)	0.434	0.031	0.016	0.294
	3	2.023(-4.939-8.984)	0.564	0.095	0.064	0.031*
	4	2.023(-4.993-9.039)	0.567	0.095	0	0.99
BMI (kg/m^2^)	1	2.998(0.393-5.602)	0.025*	0.07	0.07	0.025*
	2	2.846(0.163-5.529)	0.038*	0.074	0.004	0.606
	3	2.819(0.102-5.536)	0.042*	0.074	0.001	0.846
	4	2.814(0.079-5.55)	0.044*	0.076	0.002	0.723
Resting heart rate (bpm)	1	-1.658(-7.305-3.99)	0.56	0.005	0.005	0.56
	2	-2.818(-8.506-2.87)	0.326	0.053	0.048	0.066
	3	-2.972(-8.722-2.777)	0.306	0.057	0.004	0.601
	4	-2.976(-8.769-2.817)	0.309	0.057	0	0.887
Systolic blood pressure	1	0.479(-5.484-6.441)	0.873	0	0	0.873
	2	0.53(-5.625-6.684)	0.864	0	0	0.94
	3	0.547(-5.687-6.781)	0.861	0	0	0.956
	4	0.525(-5.731-6.781)	0.867	0.009	0.008	0.456
Diastolic blood pressure	1	-1.671(-5.152-1.811)	0.342	0.013	0.013	0.342
	2	-1.765(-5.357-1.828)	0.331	0.014	0.001	0.812
	3	-1.753(-5.391-1.886)	0.34	0.014	0	0.95
	4	-1.765(-5.418-1.888)	0.338	0.021	0.007	0.481
Natural log-transformed delta FHS score	1	0.028(-0.136-0.192)	0.734	0.002	0.002	0.734
	2	0.071(-0.091-0.233)	0.383	0.081	0.08	0.017*
	3	0.059(-0.103-0.22)	0.469	0.111	0.029	0.139
	4	0.056(-0.091-0.204)	0.45	0.27	0.159	<0.001***

Footnotes: CP= cerebral palsy; MCI=mild cognitive impairment; hs-CRP=high-sensitivity C-reactive protein; BDNF=brain-derived neurotrophic factor; #Semantic fluency (60-second animal naming); WAIS= Wechsler Adult Intelligence Scale, BMI= Body-mass index; bpm= beats per minute; FHS=Framingham heart study; 95% CI=95% confidence interval. * indicates <0.05, ** indicates <0.01, *** indicates <0.001. Model 1: no covariates, Model 2: added gender, Model 3: added years of formal education (in years), Model 4: added chronological age (in years).

In the MCI cohort, log-transformed BDNF has no significant correlation with log-transformed hs-CRP, neurocognitive measures, anthropometric, and physiological measures (Table 5B).

**Table 5B:** Associations of BDNF with biomarker, neurocognitive, and anthropometric measures MCI cohort.

**Dependent variable: Biomarkers /Neurocognitive /Anthropometric measures**	**Models**	**Independent variable: Log-transformed BDNF**				
		**β (95% CI)**	***P*-value of regression models**	***R^2^***	***R^2^* Change**	***P*-value of *R^2^* Change**
Log-transformed hs-CRP	1	0.07(-0.075-0.214)	0.337	0.017	0.017	0.337
	2	0.074(-0.072-0.221)	0.314	0.023	0.006	0.582
	3	0.074(-0.075-0.223)	0.323	0.023	0	0.997
	4	0.075(-0.077-0.226)	0.326	0.023	0	0.919
Semantic Fluency#	1	-0.928(-2.033-0.177)	0.098	0.051	0.051	0.098
	2	-0.907(-2.028-0.214)	0.111	0.053	0.002	0.72
	3	-0.778(-1.889-0.332)	0.166	0.106	0.053	0.089
	4	-0.854(-1.955-0.247)	0.126	0.146	0.04	0.132
WAIS-V Block Design	1	-2.836(-6.228-0.556)	0.099	0.05	0.05	0.099
	2	-2.321(-5.49-0.848)	0.148	0.197	0.146	0.003**
	3	-1.781(-4.806-1.244)	0.243	0.296	0.099	0.01*
	4	-2.107(-5.001-0.786)	0.15	0.374	0.079	0.016*
BMI (kg/m^2^)	1	1.058(-0.32-2.435)	0.129	0.043	0.043	0.129
	2	1.014(-0.38-2.408)	0.15	0.049	0.006	0.555
	3	0.918(-0.489-2.325)	0.196	0.068	0.019	0.311
	4	0.855(-0.559-2.268)	0.23	0.086	0.018	0.325
Resting heart rate (bpm)	1	-2.128(-5.851-1.594)	0.257	0.024	0.024	0.257
	2	-1.778(-5.445-1.89)	0.335	0.082	0.058	0.076
	3	-1.701(-5.436-2.034)	0.365	0.084	0.002	0.76
	4	-1.935(-5.653-1.783)	0.301	0.118	0.034	0.168
Systolic blood pressure	1	3.907(-3.97-11.784)	0.324	0.018	0.018	0.324
	2	4.679(-3.061-12.42)	0.231	0.081	0.063	0.065
	3	5.343(-2.424-13.11)	0.173	0.11	0.029	0.206
	4	5.798(-1.952-13.548)	0.139	0.139	0.029	0.199
Diastolic blood pressure	1	1.694(-2.393-5.781)	0.409	0.013	0.013	0.409
	2	1.948(-2.149-6.046)	0.344	0.038	0.025	0.246
	3	2.266(-1.857-6.39)	0.275	0.063	0.025	0.254
	4	1.821(-2.123-5.765)	0.358	0.167	0.104	0.016*
Natural log-transformed delta FHS score	1	0.191(-0.078-0.46)	0.161	0.037	0.037	0.161
	2	0.22(-0.042-0.482)	0.098	0.114	0.078	0.038*
	3	0.232(-0.034-0.497)	0.086	0.121	0.007	0.522
	4	0.246(-0.02-0.512)	0.07	0.145	0.024	0.243

Footnotes: CP= cerebral palsy; MCI=mild cognitive impairment; hs-CRP=high-sensitivity C-reactive protein; BDNF=brain-derived neurotrophic factor; #Semantic fluency (60-second animal naming); WAIS= Wechsler Adult Intelligence Scale, BMI= Body-mass index; bpm= beats per minute; FHS=Framingham heart study; 95% CI=95% confidence interval. * indicates <0.05, ** indicates <0.01, *** indicates <0.001. Model 1: no covariates, Model 2: added gender, Model 3: added years of formal education (in years), Model 4: added chronological age (in years).

### Sub-group analyses based on clinical characteristics of CP

Furthermore, we performed two sub-group analyses (Supplementary Tables 1–4), with one set of analyses stratified by CP type (hemiplegic versus non-hemiplegic) and another set stratified by STMS results. Regardless of the sub-group stratifications, the presence and the lack thereof of the associations in the sub-groups were very similar to those of the total sample.

## DISCUSSION

**Figure 1:**
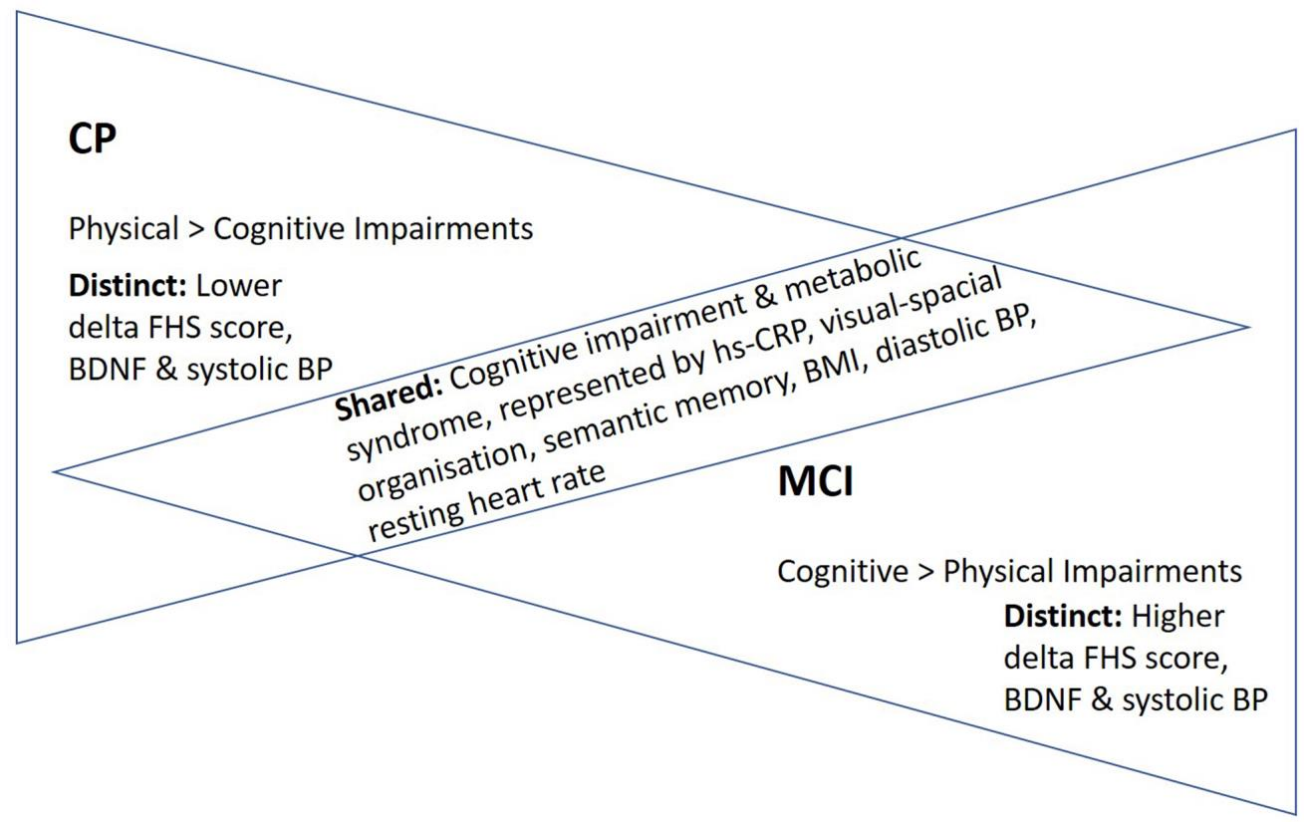
**“Inverted” disease trajectories and shared and distinct biomarkers/phenotypes between adults with CP and MCI.** A total of six examined measures were comparable between the two cohorts. Taken together these shared biomarker and phenotypes, we proposed a model of “inverted” disease trajectories between CP and MCI, with the shared biomarkers and phenotypes between them represent the “cross-road” where the pathology and phenotypes overlapped in their respective disease trajectories. Furthermore, because the mean age of the adults with CP (25 years old) was much younger than that of older adults with MCI (71 years old), we thus proposed an “accelerated aging” hypothesis, which postulates that young adults with CP have a rate of aging that is accelerated, predisposing them to have similar biological underpinning and phenotypes as older adults with MCI. Abbreviations: FHS=Framingham Heart Study; BDNF=Brain-Derived Neurotropic Factor; BP=Blood pressure; BMI=body-mass index; hs-CRP=high-sensitivity c-reactive protein.

Based on the nine measures examined in this study, there were six common biomarkers, neurocognitive, and anthropometric measures between CP and MCI, supportive of our hypothesis that these two conditions shared similar biological underpinnings and phenotypes. The commonalities in biomarker and phenotypes included hs-CRP, visual-spatial organization, semantic memory, BMI, diastolic BP, and resting heart rate. On the other hand, although lesser in comparison, there were also distinct biology and phenotype, as evidenced by natural log-transformed delta FHS score, log-transformed BDNF levels, and systolic BP (Figure 1). Next, examining if there were significant associations between the biomarkers with the phenotypes, we associated the biomarkers with the phenotypic measures. Interestingly, the natural log-transformed delta FHS score had significant associations with hs-CRP and semantic fluency in the CP cohort. In the total sample analyses, log-transformed hs-CRP was significantly associated with log-transformed BDNF and BMI, suggestive of its role in regulating neurotrophin and weight. In cohort stratified analyses, log-transformed BDNF was significantly associated with log-transformed hs-CRP and BMI in the CP cohort, suggesting that BDNF is associated with the observed inflammation and obesity in CP. For most of the associations between the measures examined in this study, chronological age was not a significant covariate, suggesting that the associations and lack thereof between the measures were not dependent on the effect of aging.

In all, these findings supported our hypothesis on common biological underpinnings between MCI and CP. These commonalities could potentially be attributed to three plausible factors, namely commonalities in pathophysiology between the two conditions, the effect of aging, and/or statistical artifacts. Since one of the common etiologies of CP is intrauterine infections [29], previous studies speculated that these infections may not have fully resolved and left persistent immunological memory similar to the effect of cytomegalovirus (CMV) on the aging immune system [21, 22], manifesting as persistently elevated low-grade inflammatory marker. In our study, we suggest that it is in the form of hs-CRP. Specifically, MCI and dementia are also preceded by unresolved immune response and persistent CMV-activated T-cells secreting inflammatory markers, leading to a higher risk of developing dementia [23, 24]. With this commonality in biomarker, we postulate that inflammation may be the “fire that started it all” in adults with CP, eventually culminating in a heightened risk of cognitive impairment. Such a systemic inflammatory phenomenon has been shown to impact a myriad of biomarkers and outcomes, including the BDNF, BMI, and cognition, which we measured and demonstrated significant associations in this study.

**Figure 2:**
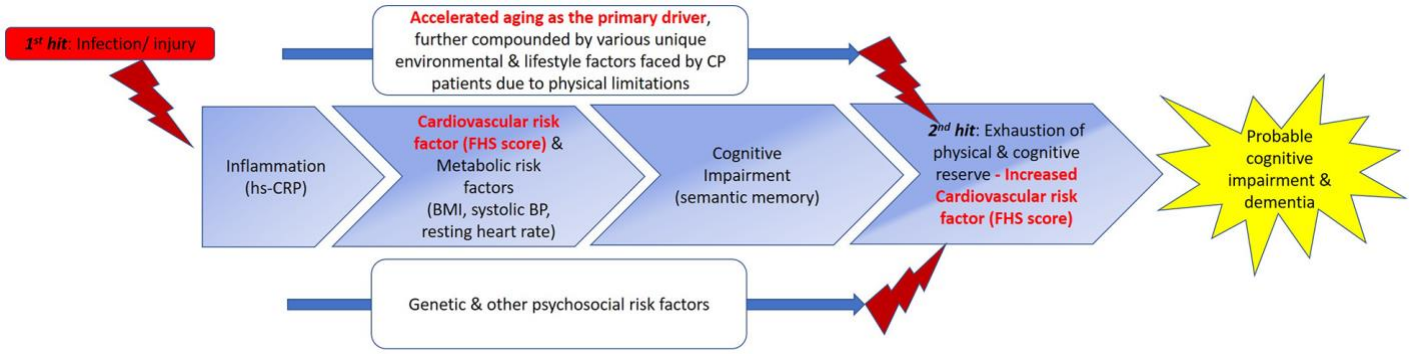
**Proposed double-hit model for the early/ “premature” development of cognitive impairment and ultimately dementia in CP through shared biomarkers and phenotypes with MCI.** We propose a double-hit model that hypothesizes the “kick-starter” effects of injury/ infections suffered at birth or during early childhood that result in persistent low-grade systemic inflammation in individuals with CP. (From left to right) With low-grade systematic inflammation mediated by hs-CRP, metabolic syndrome (MetS) could develop, as demonstrated by the association between the hs-CRP and delta FHS score in CP. MetS has been shown to be a prominent risk factor for the development of cognitive impairment, evidenced in our study by semantic memory scores in CP comparable to those of MCI, and association of delta FHS score with semantic fluency scores. The effects of accelerated aging, further compounded by various environmental, psychosocial, and lifestyle factors uniquely faced by adults with CP, due to physical limitations, further exacerbate the progression of CP to develop clinical symptoms of cognitive impairments, eventually culminating in clinical syndrome of dementia. Apart from these aforementioned factors, genetic and other psychosocial risk factors may plausibly influence the progression of this proposed continuum of dementia development by accelerating or decelerating the progression in this trajectory. Beyond what we have examined in this study, eventually, the influences of all the above-mentioned factors (main boxes and two lines) intertwine, tipping the homeostasis and eventual allostasis of the body, resulting in the progression to a phase represented by box number 4. This stage represents the second hit of our proposed double hit model, cumulating in the “breaking point”. We hypothesize that this phase is where both physical and cognitive reserve run out, causing the biomarkers, cognitive functions, and various phenotypes to further deteriorate, causing the early/ “premature” development of cognitive impairment severe enough, and coupled with the physical impairments, the adult with CP thus fulfil the clinical criteria for dementia. Based on our data, we speculate that once the reserves are exhausted in this process, CVS and metabolic risk factors play a more prominent effect (Table 4A, model 3 versus model 4, without and with aging as covariate), once aging is taken into account, delta FHS score became significantly associated with hs-CRP in patients with CP, supporting the penultimate role of aging in this trajectory. Interestingly, although BDNF levels may be lower in CP patients, symptoms of cognitive impairment have not manifested yet in CP. This could be due to the buffering from reserves [20]. But once reserves were run out (second hit and beyond), plausibly due to increased CVS risk factors, BDNF could not be further buffered and symptoms of cognitive impairment manifest. Picture adapted from Harding A, Robinson S, Crean S, Singhrao SK. “Can Better Management of Periodontal Disease Delay the Onset and Progression of Alzheimer’s Disease?” J Alzheimers Dis. 2017; 58:337-348. https://doi.org/10.3233/JAD-170046. Abbreviations: FHS=Framingham Heart Study; BDNF=Brain-Derived Neurotropic Factor; BP=Blood pressure; BMI=body-mass index; hs-CRP=high-sensitivity c-reactive protein; MetS=metabolic syndrome.

Apart from hs-CRP levels that were comparable between CP and MCI cohorts, several measures representing a range of phenotypes were also comparable between CP and MCI. They were visual-spatial organization skills, semantic memory, BMI, diastolic BP, and resting heart rate. Notably, cognitive impairment has been shown to be prevalent as individuals with CP progress into adulthood [25, 26]. Furthermore, we previously showed that metabolic syndrome is a prominent clinical characteristic of CP [8, 14, 27]. Although we did not have the measures to examine metabolic syndrome in the MCI cohort, several metabolic measures, including BMI, diastolic BP, and resting heart rate, were comparable between the two cohorts. Taken together these commonalities in biomarkers and phenotypes, we proposed a model of “inverted” disease trajectories between CP and MCI, with the common biomarkers and phenotypes between them representing the “cross-road” where the pathology and phenotypes overlapped in their respective disease trajectories (Figure 1). Because the mean age of the adults with CP (25 years old) was much younger than that of older adults with MCI (71 years old), we thus proposed an “accelerated aging” hypothesis with this model, postulating that young adults with CP have a rate of aging that is accelerated, predisposing them to have similar biological underpinning and phenotypes as older adults with MCI (Figure 2).

Furthermore, we showed that the delta FHS score in CP was significantly associated with two measures: hs-CRP and semantic memory. Although limited by the study’s cross-sectional nature, based on our preliminary findings, we proposed an aging model postulating a series of events causing the “premature” development of cognitive impairment and ultimately dementia in individuals with CP (Figure 2). Apart from these associations, we also showed that adults with CP had significantly lower delta FHS score, compared to older adults with MCI. This could potentially explain that despite many commonalities in measures between the two cohorts, cognitive impairment has yet to manifest in adults with CP. As shown in the right side of Figure 2, we propose that once the reserves are exhausted in the trajectory of aging in adults with CP, potentially caused by accelerated aging-induced increased cardiovascular risk factor (FHS score comparable to MCI), clinical symptoms of cognitive impairment will then start to manifest and ultimately leads to the development of dementia.

Our results suggest that individuals with MCI and CP have similar age-related health conditions, as shown by hs-CRP, BMI, and impairments in neurocognitive function. Thus, we further investigated the hypothesis that hs-CRP was associated with certain aging-related phenotypes. Hs-CRP was significantly correlated with BMI, in agreement with previous studies on the roles of CRP in weight and obesity. This finding thus supported the prominent roles of CRP as a shared mechanism underpinning BMI in both CP and MCI. Nonetheless, we note that the cross-sectional nature of our study limited our ability to establish any causal effects. Thus, we propose longitudinal follow-up cohorts to be established to further study this intriguing plausibility of causality. Another measure of which hs-CRP significantly associated with was BDNF. However, the association was significantly moderated by the cohort effect, suggesting differential strengths of associations that were dependent on the cohort. Hs-CRP was also significantly associated with natural log-transformed delta FHS score. Conversely, CRP had no significant correlations with the other measures. These findings suggest that CRP is but one of many pathologies common between both MCI and CP that accounts for specific phenotypes, thus highlighting the need to further explore other inflammatory biomarkers in CP, including IL-1β, IL-6, TNF-α, complement proteins, and T and B cell subpopulations.

In addition, BDNF was significantly lower in CP compared to MCI. This lower BDNF levels in adults with CP have several clinical implications; BDNF is a neurotrophic factor responsible for supporting the survival of existing neurons and encourages growth and differentiation of new neurons and synapses. It is active in the hippocampus, cerebral cortex, and basal forebrain—areas vital to learning, memory, and higher cognitive abilities. Although BDNF levels in MCI did not significantly decrease compared to healthy controls, there are significantly lower BDNF levels in patients with AD [20]. Hence, significantly lower levels of BDNF in adults with CP warrants greater attention and replication in future studies. Although we showed that BDNF was not significantly associated with neurocognitive measures in CP, studies in other populations have shown contradictory findings [28–30]; A plausible interpretation is our study was underpowered. Alternatively, BDNF may not be prominently responsible for the cognitive domains examined in CP. Another closely-related growth factor, the insulin-growth factor-1 (IGF-I), is a potential target to be examined in future study, as it has been shown to associate significantly with various cognitive domains in both healthy [31] and cognitively-impaired older adults [32, 33], establishing its role as a key biomarker in neurological condition [34]. Although it did not associate with cognitive phenotypes, the sub-group analyses suggest that in CP, BDNF was significantly associated with and thus played prominent roles in inflammation and weight in CP. With this significantly decreased BDNF levels compared with MCI, coupled with its associations with phenotypical measures, BDNF may still be a useful marker as an interventional target for adults with CP.

Lastly, the findings from the two sub-group analyses based on the clinical characteristics of CP did not differ from those of the total sample. Hence, these results suggest that the different CP subtypes, and thus the associated ID, did not affect the association and the risk of cognitive impairment in our study. Of note, although we would like to stratify the total sample by quadriplegic versus non-quadriplegic subtype, with only three subjects having a diagnosis of quadriplegic CP, this subgroup analysis was not feasible.

### Limitations

We acknowledged several limitations which present in this study, mainly conferred by the study’s pilot and exploratory nature. First, we could not completely exclude the possibility of residual confounding effects, since our study cohorts were recruited from two different countries, including participants of different ethnicities. Hence, these findings are preliminary and require validation in larger studies. Several pertinent covariates to be taken account in future comparisons, including the batch effects across the two cohorts in examining biomarkers, the *BDNF* and *APOE* genotypes, exercise, diets, intakes of supplements, and changes in medication consumption. However, such extensive controls for potential confounders would only be feasible in large cohort studies with both clinical conditions present, which is unlikely as of present. Second, there is also contention in the literature on how well blood markers reflect brain-based biomarkers in general, particularly BDNF. Conversely, there have been increasingly overwhelming evidence supporting the utility of blood-based biomarkers to examine neurological disorders. Third, also due to the pilot and exploratory nature of this study, we did not control for multiple testing. Similar practice has been adopted by other studies of pilot and exploratory nature [52, 53]. With our encouraging pilot findings, we provided strong preliminary data for future validation studies. Fourth, we did not examine the key biomarkers for Alzheimer’s dementia, such as Tau and amyloid beta. Lastly, we examined the associations between the measures cross-sectionally, hence the proposed sequence of events presented in Figure 2 required future empirical validation utilizing longitudinal cohort study. However, to our best knowledge, this is the first hypothetical comparative aging model that is backed by preliminary data postulating the connections between CP and MCI, providing encouraging impetus and supporting future pursuance in this direction. In fact, we are following up with these participants longitudinally to validate the proposed model.

### Strengths

Despite these limitations, this study made significant contributions on several aspects. To our knowledge, this study was the first to compare multiple characteristics of patients with CP and MCI directly, investigating the commonalities and differences in various biological and phenotypical measures. Furthermore, the participants were also clinically well-characterized by clinical experts in their respective fields of expertise, coupled with a number of biomarkers and clinical measures compared and contrasted across these two cohorts. The moderate sample sizes of the two cohorts also enabled us to unravel several significant associations, revealing the common and distinct biomarkers and phenotypes in these conditions, and further proposing hs-CRP as a prominent biomarker for adults with CP.

### Conclusion and future directions

In all, these preliminary findings on the common and distinct biological underpinnings and phenotypes between CP and MCI are novel and encouraging. Coupled with the fact that psychosocial interventions have been demonstrated to improve both outcomes and biomarkers in a wide range of neurological conditions, we believe this approach deserves further study. Nevertheless, due to the pilot and preliminary nature of the study, there is still limited evidence at this stage to draw definite conclusions on the common and distinct biological underpinnings and phenotypes in these two populations, or to recommend interventions in targeting them. Further validation of these findings is warranted, particularly in large-scale longitudinal follow-up cohorts recruiting participants with both CP and MCI. A number of critical future directions include taking into account of different subtypes of CP, as there could be different biomarkers associated with different etiologies characterizing the varied symptoms defining the different CP sub-types. Importantly, a prominent symptom in CP is motor dysfunction, often in the form of increased muscle tone and poor motor control. Searching for biological signatures of these symptoms could help elucidate the biological underpinnings and illuminate biological targets for individualized intervention. A life-course perspective should also be considered, with annual or bi-annual follow-ups, to validate our hypothesized effect of accelerated aging trajectory of adults with CP to developing cognitive impairment and ultimately dementia. Lastly, an examination of more comprehensive biomarkers, including nutritional status, amyloid beta and tau, and neurocognitive domains, including global cognition, are imperative to understand the complex interplay between common and distinct measures further. By comparing MCI and CP with these multi-unit examinations, future studies could shed light on how adults with CP could have increased geriatric-associated pathology and accelerated aging that may further impair function, ultimately contributing to the heightened risk of developing geriatric syndromes, earlier than their peers who were not beset with a pediatric-onset condition.

## MATERIALS AND METHODS

### Settings, study design, and participants

### Colorado site (USA): Adults with cerebral palsy (CP)

The CP cross-sectional study was approved by the Colorado Multiple Institutional Review Board (COMIRB Reference No: 14-0367) and registered with the clinical trial database (https://clinicaltrials.gov/ct2/show/NCT02137005). The study was conducted at a clinical motion analysis laboratory at the Children’s Hospital Colorado. The laboratory has a specialized team of clinicians and researchers (MDs, nurses, physical therapists, biomechanists, nurses, biogerontologists and psychologists) and is internationally accredited by the Commission for Motion Laboratory Accreditation (CMLA) (http://www.cmlainc.org/). Clinical and research staff were trained in the systematic conduct of the study procedures, such as physical examination, medical history, psychological assessments, and blood collection and composition analysis, under a standard human ethics approved protocol.

*Colorado cohort inclusion and exclusion criteria*: Participants with a confirmed diagnosis of CP were identified from an internal patient registry comprised of approximately 526 participants, aged 18 and above. Potential research participants underwent a short telephone screening survey to confirm eligibility. Participants were included in the study if they were (1) interested and able to participate in the study, (2) previous patient from the study clinic, (3) had a medical record on file at the clinical site, and (4) had mild CP by being able to walk across a 35-foot (10.6m) walkway, with or without assistive devices, at least three times. A total of 72 ambulatory participants, who passed the study screening criteria, were enrolled [35].

*CP diagnosis*: Cerebral palsy (CP) is a group of disorders that affect a person’s ability to move and maintain balance and posture. CP is a lifetime disability and the most common motor disability in childhood. CP is caused by abnormal brain development, or damage, to the developing brain that affects a person’s ability to control muscles. Many individuals with CP might also have other neurological conditions such as intellectual disability; seizures; problems with vision, hearing, or speech [36]. Although abnormal gait patterns in CP are related to posture and movement impairment, the symptoms of CP vary from person to person [37]. A person with mild CP might be able to walk independently while someone with severe CP might need lifelong care. Presently, there is limited knowledge explaining how a person with CP experience aging such as how the symptoms change over time and the types of secondary health conditions they might develop as they age [14].

*Gross motor function classification system (GMFCS)*: The Gross Motor Function Classification System (GMFCS) is a multi-level categorization tool that helps to describe varying levels of severity in people with CP [37, 38]. The GMFCS is categorized in five different levels (I, II, III, IV, V); the lower levels (I-III) correspond with milder forms of CP, while the higher levels (IV, V) indicate increased severity. The GMFCS can be used to describe all types and severity levels of CP. This classification provides both the patient and the clinician with a description of the patient’s current motor function [38].

*Cerebral palsy topographical classification*: The topographical classification of CP is used to diagnose and describe the body part(s) and side(s) that are affected by the condition [39]. Usually, these are described as 1) paresis for a weakened part and plegia/plegic for paralyzed; 2) monoplegia/monoparesis when only one limb is affected and hemiplegia/hemiparesis when the limb is significantly impaired; 3) diplegia/diparesis usually indicates when the legs are the part of the body that are severely affected; 4) hemiplegia/hemiparesis is used when the arm and leg on one side of the body are affected; 5) paraplegia/paraparesis means the lower half of the body is affected, including both legs; 6) triplegia/triparesis indicates that three limbs are affected (i.e. both arms and a leg or both legs and arm) as well as one upper and one lower extremity and the face; 7) double hemiplegia/double hemiparesis indicates all four limbs are involved, but one side of the body is more affected than the other; 8) tetraplegia/tetraparesis indicates that all four limbs are involved, but three limbs are more affected than the fourth; 9) quadriplegia/quadriparesis is used when all four limbs are involved; and 10) pentaplegia/pentaparesis means all four limbs are involved, with neck and head paralysis often accompanied by eating and breathing complications [39].

*Mild cognitive impairment (MCI) screening in cerebral palsy (CP)*: The Short Test of Mental Status (STMS) [40, 41] was used to identify mild cognitive impairment in the CP cohort. STMS has been widely used in neurological clinical settings as a brief and reliable standardize cognitive screening tool for mild cognitive impairment detection. Cognitive screening tools are commonly used in the older adult population, but many of them lack sensitivity and specificity in mild cognitive impairment (MCI) detection. In addition, MCI is under-diagnosed in adults with CP. The Short Test of Mental Status (STMS) has a higher sensitivity and specificity in detecting early cognitive deficits, such as MCI, as compared to the Mini Mental State Examination (MMSE) [42, 43]. Since the CP cohort was relatively young, the study clinical team recommended the STMS over the MMSE as a more suitable cognitive screening tool for the CP cohort.

### Singapore site (Republic of Singapore): Older adults with MCI

This study was approved by the National University of Singapore ethics committee, Institutional Review Board (NUS-IRB Reference No: B-14-110), and registered with the clinical trial database (https://clinicaltrials.gov/ct2/show/NCT02286791). The participants were older adults aged 60 and above, who have participated in the Mindfulness Awareness Practice randomized controlled trial, at a community-based research center established by the NUS Psychological Medicine department. The research nurses, research assistants, and a Ph.D. student obtained informed consent before screening for potentially eligible participants.

*Singapore cohort inclusion and exclusion criteria*: The inclusion criterion was fulfilling the operational criteria of MCI based on The Diagnostic and Statistical Manual of Mental Disorders, Fifth Edition (DSM-V) [44]. We excluded older adults with either dementia or normal aging, had a neurological or major psychiatric condition, had a terminal illness, had visual or hearing impairments, had upper and lower limb motor difficulties, and those who were participating in another intervention at the time of the screening. To derive the cognitive status of the participants, there was a two-tier procedure. First, the assessors, comprised of a team of trained research assistants and a Ph.D. candidate, administered the clinical dementia rating (CDR) and neurocognitive assessments (NCA) to all screened participants at the research center and derived at preliminary research diagnosis. Final research diagnoses of MCI were made during the study’s consensus meetings by a panel consisting of at least two consultant-ranked psychiatrists, clinical scientists, and the trained assessors who administered the tests.

*Mild cognitive impairment (MCI) diagnosis in community-dwelling older adults*: A diagnosis of amnestic MCI and non-amnestic MCI was made during the MCI cohort’s monthly consensus meeting, by a panel consisting of at least two consultant-ranked psychiatrists, clinical scientists, and the trained assessors who administered the tests. A diagnosis of amnestic MCI is mostly indicative of memory loss as the predominant symptom and is a prodromal stage of Alzheimer’s disease. On the other hand, a diagnosis of non-amnestic MCI is often associated with non-memory related cognitive impairment and is a prodromal stage of other dementia types [45].

### Overlapping measurements across both cohorts

After examining the datasets for the two cohorts, we identified nine overlapping measures that have been associated to aging and early cognitive impairment progression, which we divided into two categories, namely biomarkers (N=3) and phenotypic measurements, and sub-divided into two sub-classes: neurocognitive (N=2) and anthropometric measures (N=4).

### Biomarker measurements across both cohorts

*Bio-specimen collections*: For both cohorts, blood collections were scheduled between 9:00 and 11:00 in the morning to minimize diurnal variations. The participants stopped the consumption of foods after 10 pm the night before venipuncture. The consumption of only water was advised. The participants were advised not to exercise or perform rigorous physical activities before the collections and not to rush to the centers in the case that they were late. Blood draw via venipuncture was performed by the research nurses on the day that the participants visited the research center. The blood was kept at 4° C for a maximum of three hours before being processed in the respective laboratories.

*Biomarker pre-processing, storage, and measurements*: The blood samples were sent to the laboratory located at the University of Colorado and Singapore Immunology Network (SIgN), for the CP and MCI cohorts, respectively. Subsequently, the whole blood samples were centrifuged at 1650×g for 25 minutes at room temperature to obtain the plasma. The plasma samples were then stored at -80° C until further analyses. After sample collections from all the participants were completed, all samples were assayed on the same day and on the same plates in the respective laboratories to avoid batch effect.

Biomarker levels were examined using commercially available enzyme-linked immunosorbent assay (ELISA) kits. A total of two overlapping biomarkers were measured, namely high-sensitivity (hs)-CRP (Tecan, Männedorf, Switzerland) and BDNF (Promega Corporation, Madison, USA). All the experiments were performed as per the instructions of respective manufacturers of the kits.

*10-year Framingham heart study (FHS) measure*: We utilized the equations with recommended measures from the FHS to determine the risk percentage for the development of CVD for each subject [35]. The FHS cardiovascular (CVD) 10-year risk factor estimation for the BMI-based results were used to determine the percentage of CVD risk in both cohorts [46]. Sex, age, systolic blood pressure, BMI, information on whether the participant was a smoker, had diabetes, or was on medication for hypertension were utilized to calculate the risk percentage for CVD. Based on the same set of measures, we also derived the estimates for the general population, which were obtained from the FHS database [46, 47]. Since we were comparing two cohorts with a large age gap, we derived the delta FHS score, by subtracting each subject’s FHS individual risk score from the corresponding risk estimates for the general population. The derived “delta FHS score” was used in all the subsequent analyses and regression models. Due to its skewed nature, we performed natural log-transformed on the delta FHS score, successfully transforming it to conform to statistical normality.

### Phenotypic measurements across both cohorts

*Neurocognitive assessments (NCA)*: Cognitive functions were examined using two neurocognitive tests that have been validated in both Singapore and the United States to have good psychometric properties [48, 49]. First, the Wechsler Adult Intelligence Scale (WAIS)-V Block Design is a sub-test that is administered as part of the WAIS-V test battery, primarily measures visual-spatial and organizational processing abilities, as well as non-verbal problem-solving skills [49, 50]. As it is a timed task, it is also influenced by fine motor skills. Second, semantic verbal fluency (60-seconds Animal Naming) taps lexical knowledge and semantic memory organization [50]. Hence, for both tests, the higher the scores, the better the participant’s cognitive function [51]. First, for the Wechsler Adult Intelligence Scale (WAIS)-V Block Design, the participants are presented with nine identical cubed blocks with two surfaces of solid red, two surfaces of solid white and two surfaces that are half red and half white. The participants were required to assemble pieces of blocks to match the figures presented to them within a specific time limit. As the participants completed more figures, the more complex the subsequent figures and more time were given. Total scores were calculated after the participants failed three consecutive trials on assembling the tasks. Hence, higher scores indicate higher cognitive functioning [51]. Second, semantic verbal fluency (60-seconds Animal Naming) taps lexical knowledge and semantic memory organization [50]. Participants were required to generate certain words corresponding to a specific semantic category (in this study, animals) within a 1-minute time limit. Animal is the most frequently used category given that: (1) it is a clear semantic category across languages and cultures; (2) it is a relatively easy semantic category with only minor differences among people living in different countries, different educational systems, or belonging to different generations; and (3) it is an easy-to-administer, short, and common test included in different cognitive tests. The total numbers of words were summed up. Optimal fluency performance involves generating words within a sub-category and, when a sub-category is exhausted, switching to a new sub-category. Hence, the higher the number of words, the better the participant’s cognitive function. The tests were administered by either the trained research nurses, a Ph.D. candidate or research assistants.

*Anthropometric measures across both cohorts*: Anthropometric data were obtained from physical examinations, administered by either the research nurses or trained research assistants. Body-mass index (BMI) was calculated by body mass divided by the square of the standing height, resulting in a unit of kg/m^2^, with a BMI higher than 24.9 considered overweight. We measured systolic and diastolic blood pressure (BP) and resting heart rate using a blood pressure monitor and cuff which we secured around the participants’ left arm. We took three blood pressure measures using a medical-grade electronic vital sign monitor (Welch Allyn Spot Vital Signs Monitor, Welch Allyn, Skaneateles Falls, NY, USA).

### Statistical analyses: comparing CP and MCI participants’ outcomes

Since this is a post-hoc exploratory study, no effect size was assumed and thus no sample size calculation was performed. All measures were expressed as mean ± standard error (SE), except gender with percentage. The differences in baseline variables were examined using Student’s *t*-test, chi-square or Fisher’s exact tests according to the nature of the data. The raw values of the biomarker measurements did not fulfill the normality assumption; therefore, the raw values of the biomarkers were natural log- or log-transformed for subsequent analyses and were successfully normalized, based on dot plots, skewness, and kurtosis. We performed linear regression analyses using the dummy variable participant cohort as the independent variable, associating with the biomarkers, anthropometric, and neurocognitive measures independently in investigating aim 1. To investigate aim 2, we used the respective biomarkers as the independent variables and associated it with the other biomarkers, anthropometric and neurocognitive measures, which acted as dependent variables. All the regression models controlled for a number of relevant covariates; We performed stepwise regression analyses, with the covariates sequentially entered into the regression models. Model 1 did not control for covariates, model 2 controlled for sex and model 3 further controlled for years of formal education. In further investigating aim 3, we further controlled for age of the participants in model 4. However, for regression models shown in Tables 2, 3, multicollinearity with the cohort effect occurred, and hence age was not included. In Tables 4A, 4B, 5A, 5B, and supplementary tables, since the samples were stratified based on cohorts, no issues of multicollinearity happened, and age was thus included in the model 4. For all the models, the participants did not have to complete all the assessments to be included in the analysis and missing values were replaced by mean substitutions. All the analyses were performed using the Statistical Package for the Social Sciences (SPSS) Statistics for Windows, version 24.0 (IBM Corp., Armonk, N.Y., USA). A two-tailed p-value of <0·05 was considered statistically significant. Due to the pilot and exploratory nature of this study, we did not control for multiple testing, similar to other studies of pilot and exploratory nature [52, 53]. For all the regression models 3 presented in Table 2, statistically significant p-values represent distinct whilst statistically insignificant p-values represent commonalities in measures between CP and MCI. A recent study indicated that adults with intellectual and developmental disabilities are more likely to develop early dementia [54]. Hence, the association between CP and dementia might be driven by neurologic or intellectual comorbidities, rather than as a direct effect of CP. Since we did not collect measures for ID, we used CP subtypes as a proxy, as certain CP subtypes are associated with a higher incidence of ID. We performed two sub-group analyses, with one set of analyses stratified by CP subtypes (hemiplegic versus non-hemiplegic) and another set of analyses stratified by the Short Test of Mental Status (STMS) results (screened with MCI versus without MCI).

## Supplementary Material

Supplementary Tables
