# Supplementary material for: Commonalities in biomarkers and phenotypes between mild cognitive impairment and cerebral palsy: a pilot exploratory study
Source: Aging (Albany NY). 2021 Jan 26;13(2):1773–816. doi: 10.18632/aging.202563 (PMC7880365; doi:10.18632/aging.202563)
Supplement: Supplementary Tables [file aging-13-202563-s001.pdf]

SUPPLEMENTARY TABLES

Supplementary Table 1A. Shared and distinct biomarkers, neurocognitive, and anthropometric measures between adults with CP and MCI.

Dependent variable: Biomarkers / Neurocognitive /Anthropometric measures	Models	Independent variable: CP versus MCI cohorts				
		β (95% CI)	*P*-value of regression models	R^2	R^2 Change	*P*-value of R^2 Change
Log-transformed hs-CRP	1	0.216(0.011-0.421)	0.039*	0.052	0.052	0.039**
	2	0.231(0.021-0.44)	0.031*	0.059	0.007	0.459
	3	0.242(-0.066-0.55)	0.121	0.059	0	0.921
Log-transformed BDNF	1	4.28(3.779-4.781)	<0.001***	0.783	0.783	<0.001***
	2	4.321(3.81-4.833)	<0.001***	0.785	0.002	0.407
	3	4.149(3.4-4.899)	<0.001***	0.786	0.001	0.532
Semantic Fluency#	1	-3.462(-5.396--1.529)	0.001**	0.137	0.137	0.001**
	2	-3.29(-5.263--1.318)	0.001**	0.146	0.009	0.368
	3	-1.015(-3.825-1.795)	0.474	0.196	0.051	0.03*
WAIS-V Block Design	1	-13.064(-18.099--8.029)	<0.001***	0.25	0.25	<0.001***
	2	-12.202(-17.266--7.137)	<0.001***	0.278	0.029	0.081
	3	-3.243(-10.144-3.657)	0.352	0.379	0.1	0.001**
BMI (kg/m^2)	1	-0.51(-2.667-1.648)	0.639	0.003	0.003	0.639
	2	-0.656(-2.862-1.55)	0.556	0.009	0.006	0.493
	3	-1.709(-4.932-1.513)	0.294	0.019	0.01	0.374
Resting heart rate (bpm)	1	-7.616(-12.335--2.896)	0.002**	0.114	0.114	0.002**
	2	-7.535(-12.374--2.696)	0.003**	0.115	0	0.863
	3	-3.445(-10.437-3.548)	0.33	0.143	0.028	0.114
Systolic blood pressure	1	15.377(6.682-24.072)	0.001**	0.134	0.134	0.001**
	2	17.401(8.802-25.999)	<0.001***	0.195	0.061	0.017*
	3	21.825(9.273-34.377)	0.001**	0.204	0.009	0.338
Diastolic blood pressure	1	-2.445(-7.014-2.123)	0.29	0.014	0.014	0.29
	2	-2.083(-6.749-2.583)	0.377	0.022	0.008	0.423
	3	0.758(-6.037-7.553)	0.825	0.038	0.016	0.256
Natural log-transformed delta FHS score	1	1.86(1.511-2.209)	<0.001***	0.584	0.584	<0.001***
	2	1.94(1.594-2.285)	<0.001***	0.612	0.028	0.019**
	3	2.184(1.682-2.686)	<0.001***	0.621	0.009	0.186

Footnotes: CP= cerebral palsy; MCI=mild cognitive impairment; hs-CRP=high-sensitivity C-reactive protein; BDNF=brain-derived neurotrophic factor; #Semantic fluency (60-second animal naming); WAIS= Wechsler Adult Intelligence Scale; BMI= Body-mass index; bpm= beats per minute; FHS=Framingham heart study; 95% CI=95% confidence interval. * indicates <0.05, ** indicates <0.01, *** indicates <0.001. Model 1: no covariates, Model 2: added gender, Model 3: added years of formal education (in years).

Supplementary Table 1B. Associations of hs-CRP with biomarker, neurocognitive, and anthropometric measures.

Dependent variable: Biomarkers / Neurocognitive / Anthropometric measures	Models	Independent variable: Log-transformed hs-CRP				
		β (95% CI)	*P*-value of regression models	R^2	R^2 Change	*P*-value of R^2 Change
Log-transformed BDNF	1	1.45(0.359-2.54)	0.01*	0.08	0.08	0.01*
	2	1.475(0.388-2.562)	0.008**	0.099	0.019	0.202
	3	0.937(0.099-1.776)	0.029*	0.486	0.386	<0.001***
	4	0.428(-0.116-0.972)	0.122	0.793	0.307	<0.001***
Semantic Fluency#	1	-2.683(-4.8--0.566)	0.014*	0.074	0.074	0.014*
	2	-2.745(-4.842--0.647)	0.011*	0.104	0.03	0.108
	3	-2.135(-4.112--0.159)	0.035*	0.236	0.133	<0.001***
	4	-2.071(-4.09--0.052)	0.045*	0.238	0.001	0.716
WAIS-V Block Design	1	-5.34(-11.368-0.688)	0.082	0.037	0.037	0.082
	2	-5.607(-11.446-0.232)	0.06	0.11	0.072	0.013*
	3	-3.157(-8.113-1.8)	0.209	0.385	0.275	<0.001***
	4	-2.834(-7.884-2.215)	0.267	0.389	0.004	0.469
BMI (kg/m^2)	1	3.825(1.706-5.943)	0.001**	0.139	0.139	0.001**
	2	3.854(1.728-5.98)	0.001**	0.145	0.006	0.449
	3	3.928(1.76-6.096)	0.001**	0.147	0.002	0.684
	4	4.274(2.103-6.444)	<0.001***	0.182	0.035	0.073
Resting heart rate (bpm)	1	-4.421(-9.628-0.787)	0.095	0.034	0.034	0.095
	2	-4.497(-9.72-0.726)	0.09	0.042	0.008	0.419
	3	-3.179(-8.205-1.846)	0.212	0.149	0.107	0.002**
	4	-2.832(-7.95-2.287)	0.274	0.156	0.007	0.441
Systolic blood pressure	1	9.459(-0.19-19.107)	0.055	0.045	0.045	0.055
	2	9.196(-0.383-18.776)	0.06	0.072	0.027	0.134
	3	7.712(-1.859-17.284)	0.113	0.111	0.039	0.068
	4	5.119(-4.067-14.306)	0.271	0.217	0.105	0.002**
Diastolic blood pressure	1	0.479(-4.382-5.34)	0.845	0	0	0.845
	2	0.392(-4.474-5.259)	0.873	0.013	0.012	0.328
	3	1(-3.899-5.9)	0.686	0.04	0.027	0.143
	4	0.933(-4.075-5.941)	0.712	0.04	0	0.879
Natural log-transformed delta FHS score	1	0.833(0.292-1.375)	0.003**	0.105	0.105	0.003**
	2	0.833(0.288-1.378)	0.003**	0.105	0	0.951
	3	0.633(0.147-1.12)	0.011*	0.315	0.21	<0.001***
	4	0.37(0.009-0.73)	0.045*	0.64	0.325	<0.001***

Footnotes: CP= cerebral palsy; MCI=mild cognitive impairment; hs-CRP=high-sensitivity C-reactive protein; BDNF=brain-derived neurotrophic factor; #Semantic fluency (60-second animal naming); WAIS= Wechsler Adult Intelligence Scale, BMI= Body-mass index; bpm= beats per minute; FHS=Framingham heart study; 95% CI=95% confidence interval. * indicates <0.05, ** indicates <0.01, *** indicates <0.00. Model 1: no covariates, Model 2: added gender, Model 3: added years of formal education (in years), Model 4: added “CP VS MCI cohort”.

Supplementary Table 1C. Associations of natural log-transformed delta FHS score with biomarker, neurocognitive, and anthropometric measures for CP cohort.

Dependent variable: Biomarkers / Neurocognitive / Anthropometric measures	Models	Independent variable: Natural log-transformed delta FHS score				
		β (95% CI)	*P*-value of regression models	R^2	R^2 Change	*P*-value of R^2 Change
Log-transformed hs-CRP	1	0.352(-0.257-0.96)	0.245	0.054	0.054	0.245
	2	0.341(-0.298-0.979)	0.282	0.055	0.001	0.876
	3	0.336(-0.465-1.138)	0.394	0.055	0	0.984
	4	0.547(-0.249-1.343)	0.168	0.188	0.133	0.071
Log-transformed BDNF	1	-0.299(-0.789-0.192)	0.222	0.059	0.059	0.222
	2	-0.241(-0.744-0.263)	0.334	0.101	0.042	0.299
	3	-0.461(-1.071-0.149)	0.131	0.162	0.061	0.209
	4	-0.347(-0.976-0.283)	0.266	0.222	0.06	0.206
Semantic Fluency#	1	6.987(0.767-13.206)	0.029*	0.176	0.176	0.029*
	2	6.756(0.243-13.269)	0.043*	0.18	0.004	0.747
	3	5.394(-2.717-13.505)	0.182	0.193	0.013	0.554
	4	4.674(-3.942-13.291)	0.273	0.206	0.013	0.556
WAIS-V Block Design	1	8.31(-6.777-23.397)	0.267	0.049	0.049	0.267
	2	8.93(-6.861-24.721)	0.255	0.054	0.005	0.721
	3	2.395(-16.817-21.607)	0.799	0.111	0.057	0.236
	4	3.085(-17.462-23.633)	0.758	0.114	0.002	0.812
BMI (kg/m^2)	1	-2.211(-9.312-4.89)	0.527	0.016	0.016	0.527
	2	-2.121(-9.571-5.33)	0.562	0.017	0.001	0.912
	3	-3.007(-12.335-6.321)	0.512	0.022	0.005	0.737
	4	-2.856(-12.844-7.131)	0.559	0.022	0.001	0.915
Resting heart rate (bpm)	1	-5.655(-17.291-5.981)	0.326	0.039	0.039	0.326
	2	-3.732(-15.398-7.935)	0.515	0.123	0.084	0.143
	3	-8.36(-22.591-5.87)	0.237	0.171	0.049	0.257
	4	-7.73(-22.939-7.479)	0.303	0.175	0.003	0.769
Systolic blood pressure	1	7.626(-5.491-20.743)	0.242	0.054	0.054	0.242
	2	5.238(-7.778-18.254)	0.414	0.155	0.1	0.104
	3	7.177(-9.096-23.449)	0.371	0.161	0.007	0.674
	4	6.239(-11.129-23.607)	0.464	0.167	0.006	0.703
Diastolic blood pressure	1	3.3(-4.938-11.538)	0.417	0.027	0.027	0.417
	2	3.652(-4.969-12.273)	0.391	0.032	0.006	0.711
	3	10.346(0.746-19.946)	0.036*	0.238	0.206	0.02*
	4	8.794(-1.21-18.798)	0.082	0.279	0.04	0.279

Footnotes: CP= cerebral palsy; MCI=mild cognitive impairment; hs-CRP=high-sensitivity C-reactive protein; BDNF=brain-derived neurotrophic factor; #Semantic fluency (60-second animal naming); WAIS= Wechsler Adult Intelligence Scale, BMI= Body-mass index; bpm= beats per minute; FHS=Framingham heart study; 95% CI=95% confidence interval. * indicates <0.05, ** indicates <0.01, *** indicates <0.001. Model 1: no covariates, Model 2: added gender, Model 3: added years of formal education (in years), Model 4: added chronological age (in years).

Supplementary Table 1D. Associations of natural log-transformed delta FHS score with biomarker, neurocognitive, and anthropometric measures for MCI cohort.

Dependent variable: Biomarkers / Neurocognitive / Anthropometric measures	Models	Independent variable: Natural log-transformed delta FHS score				
		β (95% CI)	*P*-value of regression models	R^2	R^2 Change	*P*-value of R^2 Change
Log-transformed hs-CRP	1	0.073(-0.073-0.218)	0.321	0.019	0.019	0.321
	2	0.069(-0.083-0.22)	0.368	0.019	0.001	0.842
	3	0.07(-0.084-0.223)	0.367	0.02	0.001	0.851
	4	0.071(-0.086-0.227)	0.368	0.02	0	0.91
Log-transformed BDNF	1	0.193(-0.079-0.465)	0.161	0.037	0.037	0.161
	2	0.236(-0.045-0.516)	0.098	0.063	0.026	0.237
	3	0.244(-0.036-0.524)	0.086	0.084	0.021	0.281
	4	0.262(-0.022-0.546)	0.07	0.099	0.015	0.365
Semantic Fluency#	1	-0.795(-1.914-0.324)	0.16	0.037	0.037	0.16
	2	-0.933(-2.093-0.227)	0.113	0.053	0.016	0.356
	3	-0.998(-2.126-0.129)	0.082	0.125	0.073	0.045*
	4	-0.908(-2.043-0.227)	0.115	0.148	0.023	0.251
WAIS-V Block Design	1	0.974(-2.515-4.464)	0.578	0.006	0.006	0.578
	2	-0.367(-3.711-2.978)	0.827	0.164	0.158	0.003**
	3	-0.617(-3.761-2.526)	0.695	0.279	0.114	0.006**
	4	-0.137(-3.187-2.913)	0.929	0.347	0.069	0.026*
BMI (kg/m^2)	1	1.13(-0.251-2.511)	0.107	0.048	0.048	0.107
	2	1.354(-0.069-2.776)	0.062	0.075	0.027	0.225
	3	1.407(-0.008-2.822)	0.051	0.107	0.032	0.184
	4	1.546(0.132-2.96)	0.033*	0.142	0.035	0.158
Resting heart rate (bpm)	1	-1.047(-4.826-2.731)	0.581	0.006	0.006	0.581
	2	-2.086(-5.87-1.698)	0.274	0.087	0.081	0.037*
	3	-2.139(-5.959-1.68)	0.266	0.091	0.004	0.62
	4	-1.833(-5.678-2.012)	0.343	0.115	0.024	0.251
Systolic blood pressure	1	12.413(5.187-19.639)	0.001**	0.183	0.183	0.001**
	2	11.414(3.941-18.886)	0.003**	0.2	0.017	0.3
	3	11.202(3.715-18.69)	0.004**	0.215	0.016	0.319
	4	10.77(3.184-18.356)	0.006**	0.226	0.011	0.41
Diastolic blood pressure	1	-0.11(-4.246-4.026)	0.958	0	0	0.958
	2	-0.722(-4.993-3.549)	0.736	0.024	0.024	0.267
	3	-0.846(-5.123-3.432)	0.693	0.043	0.02	0.309
	4	-0.13(-4.236-3.977)	0.95	0.153	0.109	0.014*

Footnotes: CP= cerebral palsy; MCI=mild cognitive impairment; hs-CRP=high-sensitivity C-reactive protein; BDNF=brain-derived neurotrophic factor; #Semantic fluency (60-second animal naming); WAIS= Wechsler Adult Intelligence Scale, BMI= Body-mass index; bpm= beats per minute; FHS=Framingham heart study; 95% CI=95% confidence interval. * indicates <0.05, ** indicates <0.01, *** indicates <0.001. Model 1: no covariates, Model 2: added gender, Model 3: added years of formal education (in years), Model 4: added chronological age (in years).

Supplementary Table 1E. Associations of BDNF with biomarker, neurocognitive, and anthropometric measures CP cohort.

Dependent variable: Biomarkers / Neurocognitive / Anthropometric measures	Models	Independent variable: Log-transformed BDNF				
		β (95% CI)	*P*-value of regression models	R^2	R^2 Change	*P*-value of R^2 Change
Log-transformed hs-CRP	1	0.336(-0.155-0.826)	0.171	0.074	0.074	0.171
	2	0.386(-0.125-0.898)	0.132	0.098	0.025	0.427
	3	0.374(-0.149-0.897)	0.153	0.108	0.01	0.611
	4	0.277(-0.277-0.832)	0.31	0.154	0.045	0.289
Semantic Fluency#	1	2.12(-3.39-7.63)	0.436	0.025	0.025	0.436
	2	2.831(-2.877-8.538)	0.316	0.064	0.04	0.323
	3	2.43(-3.145-8.004)	0.377	0.156	0.092	0.127
	4	3.702(-2.12-9.523)	0.201	0.221	0.065	0.188
WAIS-V Block Design	1	5.207(-7.203-17.617)	0.396	0.029	0.029	0.396
	2	5.4(-7.722-18.521)	0.404	0.03	0.001	0.906
	3	4.46(-8.329-17.249)	0.478	0.128	0.099	0.12
	4	4.642(-9.261-18.545)	0.496	0.129	0	0.936
BMI (kg/m^2)	1	3.487(-2.161-9.136)	0.215	0.061	0.061	0.215
	2	3.535(-2.439-9.508)	0.234	0.061	0	0.949
	3	3.582(-2.557-9.721)	0.24	0.062	0.001	0.866
	4	3.691(-2.983-10.364)	0.264	0.063	0	0.92
Resting heart rate (bpm)	1	-0.049(-9.71-9.611)	0.992	0	0	0.992
	2	-2.137(-11.752-7.478)	0.651	0.114	0.114	0.091
	3	-2.403(-12.216-7.41)	0.617	0.128	0.013	0.557
	4	-3.817(-14.321-6.687)	0.459	0.155	0.027	0.411
Systolic blood pressure	1	-4.862(-15.658-5.935)	0.363	0.033	0.033	0.363
	2	-2.579(-13.355-8.196)	0.626	0.139	0.106	0.099
	3	-2.665(-13.74-8.41)	0.623	0.14	0.001	0.867
	4	-1.726(-13.703-10.251)	0.768	0.149	0.009	0.631
Diastolic blood pressure	1	-5.779(-12.145-0.588)	0.073	0.123	0.123	0.073
	2	-6.345(-13.013-0.323)	0.061	0.14	0.017	0.498
	3	-5.966(-12.601-0.668)	0.076	0.195	0.055	0.222
	4	-4.719(-11.74-2.302)	0.177	0.237	0.042	0.281
Natural log-transformed delta FHS score	1	-0.198(-0.523-0.127)	0.222	0.059	0.059	0.222
	2	-0.162(-0.501-0.177)	0.334	0.087	0.028	0.397
	3	-0.208(-0.484-0.067)	0.131	0.429	0.341	0.001***
	4	-0.161(-0.455-0.132)	0.266	0.453	0.025	0.33

Footnotes: CP= cerebral palsy; MCI=mild cognitive impairment; hs-CRP=high-sensitivity C-reactive protein; BDNF=brain-derived neurotrophic factor; #Semantic fluency (60-second animal naming); WAIS= Wechsler Adult Intelligence Scale, BMI= Body-mass index; bpm= beats per minute; FHS=Framingham heart study; 95% CI=95% confidence interval. * indicates <0.05, ** indicates <0.01, *** indicates <0.001. Model 1: no covariates, Model 2: added gender, Model 3: added years of formal education (in years), Model 4: added chronological age (in years).

Supplementary Table 1F. Associations of BDNF with biomarker, neurocognitive, and anthropometric measures MCI cohort.

Dependent variable: Biomarkers / Neurocognitive / Anthropometric measures	Models	Independent variable: Log-transformed BDNF				
		β (95% CI)	*P*-value of regression models	R^2	R^2 Change	*P*-value of R^2 Change
Log-transformed hs-CRP	1	0.07(-0.075-0.214)	0.337	0.017	0.017	0.337
	2	0.074(-0.072-0.221)	0.314	0.023	0.006	0.582
	3	0.074(-0.075-0.223)	0.323	0.023	0	0.997
	4	0.075(-0.077-0.226)	0.326	0.023	0	0.919
Semantic Fluency#	1	-0.928(-2.033-0.177)	0.098	0.051	0.051	0.098
	2	-0.907(-2.028-0.214)	0.111	0.053	0.002	0.72
	3	-0.778(-1.889-0.332)	0.166	0.106	0.053	0.089
	4	-0.854(-1.955-0.247)	0.126	0.146	0.04	0.132
WAIS-V Block Design	1	-2.836(-6.228-0.556)	0.099	0.05	0.05	0.099
	2	-2.321(-5.49-0.848)	0.148	0.197	0.146	0.003**
	3	-1.781(-4.806-1.244)	0.243	0.296	0.099	0.01*
	4	-2.107(-5.001-0.786)	0.15	0.374	0.079	0.016
BMI (kg/m^2)	1	1.058(-0.32-2.435)	0.129	0.043	0.043	0.129
	2	1.014(-0.38-2.408)	0.15	0.049	0.006	0.555
	3	0.918(-0.489-2.325)	0.196	0.068	0.019	0.311
	4	0.855(-0.559-2.268)	0.23	0.086	0.018	0.325
Resting heart rate (bpm)	1	-2.128(-5.851-1.594)	0.257	0.024	0.024	0.257
	2	-1.778(-5.445-1.89)	0.335	0.082	0.058	0.076
	3	-1.701(-5.436-2.034)	0.365	0.084	0.002	0.76
	4	-1.935(-5.653-1.783)	0.301	0.118	0.034	0.168
Systolic blood pressure	1	3.907(-3.97-11.784)	0.324	0.018	0.018	0.324
	2	4.679(-3.061-12.42)	0.231	0.081	0.063	0.065
	3	5.343(-2.424-13.11)	0.173	0.11	0.029	0.206
	4	5.798(-1.952-13.548)	0.139	0.139	0.029	0.199
Diastolic blood pressure	1	1.694(-2.393-5.781)	0.409	0.013	0.013	0.409
	2	1.948(-2.149-6.046)	0.344	0.038	0.025	0.246
	3	2.266(-1.857-6.39)	0.275	0.063	0.025	0.254
	4	1.821(-2.123-5.765)	0.358	0.167	0.104	0.016
Natural log-transformed delta FHS score	1	0.191(-0.078-0.46)	0.161	0.037	0.037	0.161
	2	0.22(-0.042-0.482)	0.098	0.114	0.078	0.038*
	3	0.232(-0.034-0.497)	0.086	0.121	0.007	0.522
	4	0.246(-0.02-0.512)	0.07	0.145	0.024	0.243

Footnotes: CP= cerebral palsy; MCI=mild cognitive impairment; hs-CRP=high-sensitivity C-reactive protein; BDNF=brain-derived neurotrophic factor; #Semantic fluency (60-second animal naming); WAIS= Wechsler Adult Intelligence Scale, BMI= Body-mass index; bpm= beats per minute; FHS=Framingham heart study; 95% CI=95% confidence interval. * indicates <0.05, ** indicates <0.01, *** indicates <0.001. Model 1: no covariates, Model 2: added gender, Model 3: added years of formal education (in years), Model 4: added chronological age (in years).

Supplementary Table 2A. Shared and distinct biomarkers, neurocognitive, and anthropometric measures between adults with CP and MCI.

Dependent variable: Biomarkers / Neurocognitive /Anthropometric measures	Models	Independent variable: CP versus MCI cohorts				
		β (95% CI)	*P*-value of regression models	R^2	R^2 Change	*P*-value of R^2 Change
Log-transformed hs-CRP	1	0.235(0.037-0.434)	0.021*	0.053	0.053	0.021*
	2	0.224(0.019-0.43)	0.033*	0.055	0.002	0.664
	3	0.21(-0.106-0.527)	0.191	0.055	0	0.907
Log-transformed BDNF	1	4.314(3.812-4.815)	<0.001***	0.748	0.748	<0.001***
	2	4.328(3.809-4.848)	<0.001***	0.748	0	0.818
	3	4.025(3.229-4.821)	<0.001***	0.751	0.003	0.32
Semantic Fluency#	1	-4.627(-6.442--2.812)	<0.001***	0.207	0.207	<0.001***
	2	-4.795(-6.67--2.919)	<0.001***	0.211	0.004	0.467
	3	-2.818(-5.656-0.02)	0.052	0.238	0.026	0.071
WAIS-V Block Design	1	-4.128(-8.73-0.475)	0.078	0.031	0.031	0.078
	2	-3.947(-8.714-0.821)	0.104	0.032	0.001	0.757
	3	5.057(-1.875-11.989)	0.151	0.137	0.104	0.001**
BMI (kg/m^2)	1	0.608(-1.153-2.37)	0.495	0.005	0.005	0.495
	2	0.321(-1.489-2.131)	0.726	0.022	0.017	0.197
	3	-0.213(-2.996-2.57)	0.88	0.024	0.003	0.616
Resting heart rate (bpm)	1	-7.843(-12.293--3.394)	0.001**	0.111	0.111	0.001**
	2	-7.581(-12.186--2.976)	0.002**	0.113	0.002	0.643
	3	-5.223(-12.284-1.838)	0.145	0.12	0.007	0.384
Systolic blood pressure	1	14.778(7.701-21.855)	<0.001***	0.149	0.149	<0.001***
	2	15.413(8.098-22.728)	<0.001***	0.153	0.004	0.48
	3	23.25(12.187-34.313)	<0.001***	0.183	0.029	0.066
Diastolic blood pressure	1	-4.649(-8.384--0.913)	0.015*	0.059	0.059	0.015*
	2	-4.228(-8.083--0.373)	0.032*	0.066	0.008	0.375
	3	0.274(-5.537-6.085)	0.926	0.105	0.038	0.045*
Natural log-transformed delta FHS score	1	1.803(1.506-2.101)	<0.001***	0.597	0.597	<0.001***
	2	1.895(1.596-2.193)	<0.001***	0.621	0.025	0.014*
	3	2.048(1.59-2.506)	<0.001***	0.624	0.003	0.383

Footnotes: CP= cerebral palsy; MCI=mild cognitive impairment; hs-CRP=high-sensitivity C-reactive protein; BDNF=brain-derived neurotrophic factor; #Semantic fluency (60-second animal naming); WAIS= Wechsler Adult Intelligence Scale; BMI= Body-mass index; bpm= beats per minute; FHS=Framingham heart study; 95% CI=95% confidence interval. * indicates <0.05, ** indicates <0.01, *** indicates <0.001. Model 1: no covariates, Model 2: added gender, Model 3: added years of formal education (in years).

Supplementary Table 2B. Associations of hs-CRP with biomarker, neurocognitive, and anthropometric measures.

Dependent variable: Biomarkers / Neurocognitive / Anthropometric measures	Models	Independent variable: Log-transformed hs-CRP				
		β (95% CI)	*P*-value of regression models	R^2	R^2 Change	*P*-value of R^2 Change
Log-transformed BDNF	1	1.339(0.396-2.283)	0.006**	0.075	0.075	0.006**
	2	1.256(0.318-2.194)	0.009**	0.105	0.03	0.075
	3	0.715(0.008-1.423)	0.048*	0.51	0.405	<0.001***
	4	0.382(-0.124-0.888)	0.137	0.757	0.247	<0.001***
Semantic Fluency#	1	-0.927(-2.919-1.064)	0.358	0.009	0.009	0.358
	2	-0.89(-2.9-1.12)	0.382	0.01	0.001	0.709
	3	-0.121(-1.956-1.714)	0.896	0.207	0.197	<0.001***
	4	0.119(-1.706-1.944)	0.897	0.238	0.031	0.053
WAIS-V Block Design	1	-1.625(-6.204-2.953)	0.483	0.005	0.005	0.483
	2	-1.476(-6.09-3.138)	0.527	0.009	0.004	0.512
	3	-0.169(-4.611-4.274)	0.94	0.118	0.108	0.001**
	4	-0.607(-5.064-3.85)	0.787	0.137	0.019	0.146
BMI (kg/m^2)	1	2.666(1.017-4.315)	0.002**	0.095	0.095	0.002**
	2	2.57(0.916-4.223)	0.003**	0.108	0.013	0.239
	3	2.555(0.868-4.242)	0.003**	0.108	0	0.92
	4	2.62(0.911-4.328)	0.003**	0.111	0.003	0.575
Resting heart rate (bpm)	1	-4.367(-8.915-0.181)	0.06	0.036	0.036	0.06
	2	-4.126(-8.693-0.441)	0.076	0.047	0.011	0.287
	3	-3.064(-7.548-1.421)	0.178	0.117	0.07	0.007**
	4	-2.67(-7.179-1.84)	0.243	0.133	0.015	0.196
Systolic blood pressure	1	6.495(-0.921-13.912)	0.085	0.03	0.03	0.085
	2	6.449(-1.042-13.939)	0.091	0.03	0	0.9
	3	5.424(-2.12-12.968)	0.157	0.055	0.025	0.116
	4	3.521(-3.558-10.601)	0.326	0.191	0.136	<0.001***
Diastolic blood pressure	1	-0.904(-4.679-2.87)	0.635	0.002	0.002	0.635
	2	-0.646(-4.421-3.129)	0.735	0.022	0.019	0.168
	3	0.297(-3.387-3.981)	0.873	0.105	0.083	0.004**
	4	0.279(-3.459-4.016)	0.883	0.105	0	0.942
Natural log-transformed delta FHS score	1	0.652(0.211-1.092)	0.004**	0.081	0.081	0.004**
	2	0.65(0.205-1.095)	0.005**	0.081	0	0.935
	3	0.443(0.062-0.825)	0.023*	0.351	0.27	<0.001***
	4	0.275(-0.014-0.564)	0.062	0.638	0.286	<0.001***

Footnotes: CP= cerebral palsy; MCI=mild cognitive impairment; hs-CRP=high-sensitivity C-reactive protein; BDNF=brain-derived neurotrophic factor; #Semantic fluency (60-second animal naming); WAIS= Wechsler Adult Intelligence Scale, BMI= Body-mass index; bpm= beats per minute; FHS=Framingham heart study; 95% CI=95% confidence interval. * indicates <0.05, ** indicates <0.01, *** indicates <0.00. Model 1: no covariates, Model 2: added gender, Model 3: added years of formal education (in years), Model 4: added “CP VS MCI cohort”.

Supplementary Table 2C. Associations of natural log-transformed delta FHS score with biomarker, neurocognitive, and anthropometric measures for CP cohort.

Dependent variable: Biomarkers / Neurocognitive / Anthropometric measures	Models	Independent variable: Natural log-transformed delta FHS score				
		β (95% CI)	*P*-value of regression models	R^2	R^2 Change	*P*-value of R^2 Change
Log-transformed hs-CRP	1	0.138(-0.431-0.706)	0.628	0.006	0.006	0.628
	2	0.236(-0.358-0.83)	0.427	0.035	0.029	0.268
	3	0.214(-0.395-0.823)	0.483	0.039	0.004	0.667
	4	0.44(-0.241-1.121)	0.199	0.086	0.047	0.16
Log-transformed BDNF	1	0.268(-0.171-0.706)	0.225	0.034	0.034	0.225
	2	0.377(-0.074-0.828)	0.099	0.092	0.058	0.108
	3	0.415(-0.043-0.873)	0.074	0.113	0.021	0.334
	4	0.371(-0.154-0.895)	0.161	0.116	0.003	0.715
Semantic Fluency#	1	4.009(-1.369-9.387)	0.14	0.05	0.05	0.14
	2	5.071(-0.526-10.667)	0.075	0.086	0.036	0.207
	3	5.373(-0.355-11.1)	0.065	0.094	0.008	0.539
	4	7.065(0.602-13.529)	0.033*	0.122	0.028	0.265
WAIS-V Block Design	1	-9.992(-22.683-2.699)	0.12	0.055	0.055	0.12
	2	-7.946(-21.239-5.347)	0.234	0.079	0.024	0.304
	3	-6.675(-20.14-6.791)	0.323	0.106	0.027	0.273
	4	-7.544(-22.971-7.882)	0.329	0.107	0.001	0.809
BMI (kg/m^2)	1	1.711(-3.105-6.528)	0.478	0.012	0.012	0.478
	2	2.685(-2.323-7.693)	0.285	0.051	0.039	0.196
	3	3.033(-2.076-8.142)	0.237	0.066	0.015	0.428
	4	2.521(-3.326-8.367)	0.389	0.069	0.003	0.707
Resting heart rate (bpm)	1	2.6(-9.064-14.264)	0.655	0.005	0.005	0.655
	2	4.517(-7.694-16.728)	0.46	0.031	0.026	0.294
	3	4.639(-7.913-17.191)	0.46	0.031	0	0.91
	4	3.81(-10.569-18.19)	0.595	0.033	0.001	0.805
Systolic blood pressure	1	-0.614(-12.474-11.246)	0.917	0	0	0.917
	2	1.821(-10.502-14.143)	0.767	0.041	0.041	0.189
	3	2.075(-10.586-14.736)	0.742	0.042	0.001	0.814
	4	0.915(-13.579-15.408)	0.899	0.045	0.003	0.731
Diastolic blood pressure	1	5.812(-0.681-12.305)	0.078	0.07	0.07	0.078
	2	6.38(-0.482-13.243)	0.068	0.077	0.007	0.579
	3	7.494(0.741-14.246)	0.03*	0.155	0.078	0.059
	4	9.588(1.98-17.196)	0.015*	0.184	0.029	0.241

Footnotes: CP= cerebral palsy; MCI=mild cognitive impairment; hs-CRP=high-sensitivity C-reactive protein; BDNF=brain-derived neurotrophic factor; #Semantic fluency (60-second animal naming); WAIS= Wechsler Adult Intelligence Scale, BMI= Body-mass index; bpm= beats per minute; FHS=Framingham heart study; 95% CI=95% confidence interval. * indicates <0.05, ** indicates <0.01, *** indicates <0.001. Model 1: no covariates, Model 2: added gender, Model 3: added years of formal education (in years), Model 4: added chronological age (in years).

Supplementary Table 2D. Associations of natural log-transformed delta FHS score with biomarker, neurocognitive, and anthropometric measures for MCI cohort.

Dependent variable: Biomarkers / Neurocognitive / Anthropometric measures	Models	Independent variable: Natural log-transformed delta FHS score				
		β (95% CI)	*P*-value of regression models	R^2	R^2 Change	*P*-value of R^2 Change
Log-transformed hs-CRP	1	0.073(-0.073-0.218)	0.321	0.019	0.019	0.321
	2	0.069(-0.083-0.22)	0.368	0.019	0.001	0.842
	3	0.07(-0.084-0.223)	0.367	0.02	0.001	0.851
	4	0.071(-0.086-0.227)	0.368	0.02	0	0.91
Log-transformed BDNF	1	0.193(-0.079-0.465)	0.161	0.037	0.037	0.161
	2	0.236(-0.045-0.516)	0.098	0.063	0.026	0.237
	3	0.244(-0.036-0.524)	0.086	0.084	0.021	0.281
	4	0.262(-0.022-0.546)	0.07	0.099	0.015	0.365
Semantic Fluency#	1	-0.795(-1.914-0.324)	0.16	0.037	0.037	0.16
	2	-0.933(-2.093-0.227)	0.113	0.053	0.016	0.356
	3	-0.998(-2.126-0.129)	0.082	0.125	0.073	0.045*
	4	-0.908(-2.043-0.227)	0.115	0.148	0.023	0.251
WAIS-V Block Design	1	0.974(-2.515-4.464)	0.578	0.006	0.006	0.578
	2	-0.367(-3.711-2.978)	0.827	0.164	0.158	0.003**
	3	-0.617(-3.761-2.526)	0.695	0.279	0.114	0.006**
	4	-0.137(-3.187-2.913)	0.929	0.347	0.069	0.026*
BMI (kg/m^2)	1	1.13(-0.251-2.511)	0.107	0.048	0.048	0.107
	2	1.354(-0.069-2.776)	0.062	0.075	0.027	0.225
	3	1.407(-0.008-2.822)	0.051	0.107	0.032	0.184
	4	1.546(0.132-2.96)	0.033*	0.142	0.035	0.158
Resting heart rate (bpm)	1	-1.047(-4.826-2.731)	0.581	0.006	0.006	0.581
	2	-2.086(-5.87-1.698)	0.274	0.087	0.081	0.037*
	3	-2.139(-5.959-1.68)	0.266	0.091	0.004	0.62
	4	-1.833(-5.678-2.012)	0.343	0.115	0.024	0.251
Systolic blood pressure	1	12.413(5.187-19.639)	0.001**	0.183	0.183	0.001**
	2	11.414(3.941-18.886)	0.003**	0.2	0.017	0.3
	3	11.202(3.715-18.69)	0.004**	0.215	0.016	0.319
	4	10.77(3.184-18.356)	0.006**	0.226	0.011	0.41
Diastolic blood pressure	1	-0.11(-4.246-4.026)	0.958	0	0	0.958
	2	-0.722(-4.993-3.549)	0.736	0.024	0.024	0.267
	3	-0.846(-5.123-3.432)	0.693	0.043	0.02	0.309
	4	-0.13(-4.236-3.977)	0.95	0.153	0.109	0.014**

Footnotes: CP= cerebral palsy; MCI=mild cognitive impairment; hs-CRP=high-sensitivity C-reactive protein; BDNF=brain-derived neurotrophic factor; #Semantic fluency (60-second animal naming); WAIS= Wechsler Adult Intelligence Scale, BMI= Body-mass index; bpm= beats per minute; FHS=Framingham heart study; 95% CI=95% confidence interval. * indicates <0.05, ** indicates <0.01, *** indicates <0.001. Model 1: no covariates, Model 2: added gender, Model 3: added years of formal education (in years), Model 4: added chronological age (in years).

Supplementary Table 2E. Associations of BDNF with biomarker, neurocognitive, and anthropometric measures CP cohort.

Dependent variable: Biomarkers / Neurocognitive / Anthropometric measures	Models	Independent variable: Log-transformed BDNF				
		β (95% CI)	*P*-value of regression models	R^2	R^2 Change	*P*-value of R^2 Change
Log-transformed hs-CRP	1	0.434(0.065-0.804)	0.022*	0.116	0.116	0.022
	2	0.416(0.037-0.794)	0.032*	0.122	0.007	0.573
	3	0.431(0.049-0.813)	0.028*	0.137	0.014	0.416
	4	0.479(0.095-0.863)	0.016*	0.178	0.041	0.165
Semantic Fluency#	1	0.381(-3.421-4.184)	0.841	0.001	0.001	0.841
	2	0.139(-3.748-4.025)	0.943	0.013	0.012	0.476
	3	0.083(-3.869-4.036)	0.966	0.015	0.002	0.78
	4	0.124(-3.945-4.193)	0.951	0.015	0	0.91
WAIS-V Block Design	1	-1.237(-10.232-7.758)	0.783	0.002	0.002	0.783
	2	-2.426(-11.432-6.58)	0.59	0.054	0.052	0.136
	3	-3.032(-12.001-5.938)	0.499	0.094	0.041	0.183
	4	-2.928(-12.161-6.306)	0.525	0.095	0	0.899
BMI (kg/m^2)	1	3.15(-0.048-6.347)	0.053	0.084	0.084	0.053
	2	2.942(-0.325-6.21)	0.076	0.096	0.011	0.469
	3	2.87(-0.449-6.189)	0.088	0.1	0.004	0.665
	4	2.687(-0.714-6.089)	0.118	0.108	0.008	0.545
Resting heart rate (bpm)	1	-2.952(-10.961-5.057)	0.461	0.013	0.013	0.461
	2	-3.678(-11.814-4.457)	0.367	0.037	0.024	0.31
	3	-3.71(-11.991-4.572)	0.371	0.037	0	0.939
	4	-4.264(-12.732-4.205)	0.315	0.05	0.013	0.462
Systolic blood pressure	1	4.316(-3.754-12.386)	0.287	0.026	0.026	0.287
	2	3.504(-4.669-11.677)	0.392	0.056	0.029	0.259
	3	3.463(-4.856-11.782)	0.405	0.056	0	0.922
	4	3.223(-5.332-11.778)	0.451	0.058	0.002	0.751
Diastolic blood pressure	1	0.913(-3.721-5.548)	0.693	0.004	0.004	0.693
	2	0.942(-3.824-5.708)	0.692	0.004	0	0.945
	3	0.599(-4.132-5.33)	0.8	0.053	0.049	0.153
	4	0.617(-4.254-5.488)	0.799	0.053	0	0.966
Natural log-transformed delta FHS score	1	0.127(-0.081-0.336)	0.225	0.034	0.034	0.225
	2	0.169(-0.033-0.37)	0.099	0.147	0.113	0.023*
	3	0.182(-0.019-0.383)	0.074	0.183	0.036	0.188
	4	0.131(-0.054-0.316)	0.161	0.343	0.16	0.003**

Footnotes: CP= cerebral palsy; MCI=mild cognitive impairment; hs-CRP=high-sensitivity C-reactive protein; BDNF=brain-derived neurotrophic factor; #Semantic fluency (60-second animal naming); WAIS= Wechsler Adult Intelligence Scale, BMI= Body-mass index; bpm= beats per minute; FHS=Framingham heart study; 95% CI=95% confidence interval. * indicates <0.05, ** indicates <0.01, *** indicates <0.001. Model 1: no covariates, Model 2: added gender, Model 3: added years of formal education (in years), Model 4: added chronological age (in years).

Supplementary Table 2F. Associations of BDNF with biomarker, neurocognitive, and anthropometric measures MCI cohort.

Dependent variable: Biomarkers / Neurocognitive / Anthropometric measures	Models	Independent variable: Log-transformed BDNF				
		β (95% CI)	*P*-value of regression models	R^2	R^2 Change	*P*-value of R^2 Change
Log-transformed hs-CRP	1	0.07(-0.075-0.214)	0.337	0.017	0.017	0.337
	2	0.074(-0.072-0.221)	0.314	0.023	0.006	0.582
	3	0.074(-0.075-0.223)	0.323	0.023	0	0.997
	4	0.075(-0.077-0.226)	0.326	0.023	0	0.919
Semantic Fluency#	1	-0.928(-2.033-0.177)	0.098	0.051	0.051	0.098
	2	-0.907(-2.028-0.214)	0.111	0.053	0.002	0.72
	3	-0.778(-1.889-0.332)	0.166	0.106	0.053	0.089
	4	-0.854(-1.955-0.247)	0.126	0.146	0.04	0.132
WAIS-V Block Design	1	-2.836(-6.228-0.556)	0.099	0.05	0.05	0.099
	2	-2.321(-5.49-0.848)	0.148	0.197	0.146	0.003**
	3	-1.781(-4.806-1.244)	0.243	0.296	0.099	0.01*
	4	-2.107(-5.001-0.786)	0.15	0.374	0.079	0.016
BMI (kg/m^2)	1	1.058(-0.32-2.435)	0.129	0.043	0.043	0.129
	2	1.014(-0.38-2.408)	0.15	0.049	0.006	0.555
	3	0.918(-0.489-2.325)	0.196	0.068	0.019	0.311
	4	0.855(-0.559-2.268)	0.23	0.086	0.018	0.325
Resting heart rate (bpm)	1	-2.128(-5.851-1.594)	0.257	0.024	0.024	0.257
	2	-1.778(-5.445-1.89)	0.335	0.082	0.058	0.076
	3	-1.701(-5.436-2.034)	0.365	0.084	0.002	0.76
	4	-1.935(-5.653-1.783)	0.301	0.118	0.034	0.168
Systolic blood pressure	1	3.907(-3.97-11.784)	0.324	0.018	0.018	0.324
	2	4.679(-3.061-12.42)	0.231	0.081	0.063	0.065
	3	5.343(-2.424-13.11)	0.173	0.11	0.029	0.206
	4	5.798(-1.952-13.548)	0.139	0.139	0.029	0.199
Diastolic blood pressure	1	1.694(-2.393-5.781)	0.409	0.013	0.013	0.409
	2	1.948(-2.149-6.046)	0.344	0.038	0.025	0.246
	3	2.266(-1.857-6.39)	0.275	0.063	0.025	0.254
	4	1.821(-2.123-5.765)	0.358	0.167	0.104	0.016*
Natural log-transformed delta FHS score	1	0.191(-0.078-0.46)	0.161	0.037	0.037	0.161
	2	0.22(-0.042-0.482)	0.098	0.114	0.078	0.038*
	3	0.232(-0.034-0.497)	0.086	0.121	0.007	0.522
	4	0.246(-0.02-0.512)	0.07	0.145	0.024	0.243

Footnotes: CP= cerebral palsy; MCI=mild cognitive impairment; hs-CRP=high-sensitivity C-reactive protein; BDNF=brain-derived neurotrophic factor; #Semantic fluency (60-second animal naming); WAIS= Wechsler Adult Intelligence Scale, BMI= Body-mass index; bpm= beats per minute; FHS=Framingham heart study; 95% CI=95% confidence interval. * indicates <0.05, ** indicates <0.01, *** indicates <0.001. Model 1: no covariates, Model 2: added gender, Model 3: added years of formal education (in years), Model 4: added chronological age (in years).

Supplementary Table 3A. Shared and distinct biomarkers, neurocognitive, and anthropometric measures between adults with CP and MCI.

Dependent variable: Biomarkers / Neurocognitive / Anthropometric measures	Models	Independent variable: CP versus MCI cohorts				
		β (95% CI)	*P*-value of regression models	R^2	R^2 Change	*P*-value of R^2 Change
Log-transformed hs-CRP	1	0.095(-0.136-0.326)	0.414	0.009	0.009	0.414
	2	0.1(-0.136-0.336)	0.4	0.01	0.001	0.804
	3	0.079(-0.257-0.415)	0.64	0.011	0	0.86
Log-transformed BDNF	1	4.264(3.643-4.884)	<0.001***	0.726	0.726	<0.001***
	2	4.294(3.66-4.928)	<0.001***	0.727	0.001	0.593
	3	4.135(3.235-5.036)	<0.001***	0.728	0.001	0.62
Semantic Fluency#	1	-9.381(-11.288--7.474)	<0.001***	0.575	0.575	<0.001***
	2	-9.31(-11.26--7.36)	<0.001***	0.576	0.001	0.686
	3	-7.92(-10.654--5.185)	<0.001***	0.589	0.012	0.156
WAIS-V Block Design	1	-18.187(-24.309--12.064)	<0.001***	0.331	0.331	<0.001***
	2	-17.747(-23.987--11.506)	<0.001***	0.337	0.006	0.433
	3	-7.974(-16.211-0.263)	0.058	0.429	0.093	0.001**
BMI (kg/m^2)	1	0.865(-1.033-2.763)	0.367	0.011	0.011	0.367
	2	0.794(-1.147-2.736)	0.417	0.014	0.002	0.687
	3	-0.466(-3.195-2.263)	0.734	0.038	0.024	0.196
Resting heart rate (bpm)	1	-6.015(-11.401--0.63)	0.029*	0.065	0.065	0.029*
	2	-5.493(-10.963--0.024)	0.049	0.08	0.015	0.29
	3	-2.434(-10.148-5.281)	0.531	0.097	0.016	0.267
Systolic blood pressure	1	12.975(2.552-23.397)	0.015*	0.08	0.08	0.015*
	2	14.049(3.475-24.622)	0.01*	0.097	0.017	0.26
	3	23.18(8.455-37.906)	0.002**	0.135	0.038	0.084
Diastolic blood pressure	1	-5.31(-10.884-0.264)	0.062	0.048	0.048	0.062
	2	-5.112(-10.813-0.588)	0.078	0.05	0.002	0.699
	3	-1.667(-9.695-6.362)	0.68	0.07	0.02	0.23
Natural log-transformed delta FHS score	1	1.893(1.504-2.283)	<0.001***	0.569	0.569	<0.001***
	2	1.972(1.587-2.357)	<0.001***	0.599	0.03	0.025**
	3	2.147(1.603-2.691)	<0.001***	0.604	0.005	0.367

Footnotes: CP= cerebral palsy; MCI=mild cognitive impairment; hs-CRP=high-sensitivity C-reactive protein; BDNF=brain-derived neurotrophic factor; #Semantic fluency (60-second animal naming); WAIS= Wechsler Adult Intelligence Scale; BMI= Body-mass index; bpm= beats per minute; FHS=Framingham heart study; 95% CI=95% confidence interval. * indicates <0.05, ** indicates <0.01, *** indicates <0.001. Model 1: no covariates, Model 2: added gender, Model 3: added years of formal education (in years).

Supplementary Table 3B. Associations of hs-CRP with biomarker, neurocognitive, and anthropometric measures.

Dependent variable: Biomarkers / Neurocognitive / Anthropometric measures	Models	Independent variable: Log-transformed hs-CRP				
		β (95% CI)	*P*-value of regression models	R^2	R^2 Change	*P*-value of R^2 Change
Log-transformed BDNF	1	0.638(-0.562-1.838)	0.293	0.016	0.016	0.293
	2	0.645(-0.554-1.845)	0.287	0.03	0.014	0.31
	3	0.376(-0.577-1.328)	0.434	0.402	0.372	<0.001***
	4	0.21(-0.437-0.856)	0.52	0.729	0.327	<0.001***
Semantic Fluency#	1	-1.909(-4.862-1.043)	0.201	0.023	0.023	0.201
	2	-1.934(-4.865-0.997)	0.192	0.051	0.028	0.153
	3	-1.29(-3.646-1.067)	0.279	0.4	0.349	<0.001***
	4	-0.973(-2.929-0.982)	0.324	0.595	0.194	<0.001***
WAIS-V Block Design	1	-3.795(-11.381-3.791)	0.322	0.014	0.014	0.322
	2	-3.863(-11.379-3.653)	0.309	0.046	0.032	0.129
	3	-2.195(-8.207-3.817)	0.469	0.403	0.357	<0.001***
	4	-1.88(-7.794-4.035)	0.528	0.433	0.03	0.064
BMI (kg/m^2)	1	2.334(0.465-4.203)	0.015*	0.08	0.08	0.015*
	2	2.341(0.463-4.218)	0.015*	0.085	0.005	0.544
	3	2.231(0.357-4.106)	0.02*	0.109	0.024	0.181
	4	2.257(0.369-4.145)	0.02*	0.112	0.003	0.628
Resting heart rate (bpm)	1	-1.719(-7.39-3.953)	0.548	0.005	0.005	0.548
	2	-1.766(-7.399-3.868)	0.534	0.033	0.028	0.16
	3	-1.252(-6.765-4.262)	0.652	0.094	0.061	0.034*
	4	-1.157(-6.706-4.392)	0.679	0.099	0.005	0.55
Systolic blood pressure	1	9.816(-1.029-20.66)	0.075	0.044	0.044	0.075
	2	9.775(-1.119-20.669)	0.078	0.049	0.005	0.527
	3	9.547(-1.451-20.546)	0.088	0.053	0.003	0.634
	4	8.64(-1.757-19.038)	0.102	0.169	0.116	0.003**
Diastolic blood pressure	1	2.236(-3.572-8.044)	0.445	0.008	0.008	0.445
	2	2.212(-3.619-8.043)	0.452	0.015	0.007	0.488
	3	2.756(-2.942-8.454)	0.338	0.08	0.065	0.03*
	4	2.833(-2.909-8.574)	0.328	0.083	0.003	0.641
Natural log-transformed delta FHS score	1	0.476(-0.12-1.071)	0.116	0.035	0.035	0.116
	2	0.475(-0.125-1.074)	0.119	0.036	0.001	0.776
	3	0.368(-0.16-0.895)	0.169	0.269	0.234	<0.001***
	4	0.282(-0.104-0.668)	0.15	0.616	0.347	<0.001***

Footnotes: CP= cerebral palsy; MCI=mild cognitive impairment; hs-CRP=high-sensitivity C-reactive protein; BDNF=brain-derived neurotrophic factor; #Semantic fluency (60-second animal naming); WAIS= Wechsler Adult Intelligence Scale, BMI= Body-mass index; bpm= beats per minute; FHS=Framingham heart study; 95% CI=95% confidence interval. * indicates <0.05, ** indicates <0.01, *** indicates <0.00. Model 1: no covariates, Model 2: added gender, Model 3: added years of formal education (in years), Model 4: added “CP VS MCI cohort”.

Supplementary Table 3C. Associations of natural log-transformed delta FHS score with biomarker, neurocognitive, and anthropometric measures for CP cohort.

Dependent variable: Biomarkers / Neurocognitive / Anthropometric measures	Models	Independent variable: Natural log-transformed delta FHS score				
		β (95% CI)	*P*-value of regression models	R^2	R^2 Change	*P*-value of R^2 Change
Log-transformed hs-CRP	1	0.023(-0.829-0.875)	0.955	0	0	0.955
	2	0.055(-0.884-0.993)	0.903	0.003	0.003	0.834
	3	0.076(-0.904-1.056)	0.87	0.013	0.01	0.716
	4	0.124(-0.975-1.223)	0.811	0.018	0.005	0.802
Log-transformed BDNF	1	0.341(-0.496-1.178)	0.401	0.045	0.045	0.401
	2	0.514(-0.365-1.393)	0.232	0.134	0.089	0.233
	3	0.463(-0.43-1.355)	0.285	0.19	0.056	0.343
	4	0.29(-0.673-1.253)	0.527	0.254	0.064	0.31
Semantic Fluency#	1	-0.07(-7.734-7.594)	0.985	0	0	0.985
	2	-0.137(-8.59-8.315)	0.973	0	0	0.961
	3	0.109(-8.691-8.909)	0.979	0.017	0.016	0.637
	4	1.765(-7.752-11.281)	0.695	0.09	0.073	0.325
WAIS-V Block Design	1	-4.137(-29.895-21.62)	0.738	0.007	0.007	0.738
	2	2.267(-24.15-28.684)	0.857	0.142	0.134	0.146
	3	0.964(-26.111-28.039)	0.94	0.182	0.04	0.421
	4	-2.91(-32.681-26.861)	0.836	0.217	0.035	0.458
BMI (kg/m^2)	1	-2.027(-6.317-2.264)	0.332	0.059	0.059	0.332
	2	-2.704(-7.306-1.897)	0.23	0.11	0.051	0.367
	3	-2.804(-7.613-2.005)	0.232	0.118	0.008	0.727
	4	-2.432(-7.803-2.939)	0.346	0.129	0.011	0.69
Resting heart rate (bpm)	1	-0.553(-16.364-15.259)	0.942	0	0	0.942
	2	2.097(-14.797-18.992)	0.795	0.062	0.062	0.337
	3	1.344(-16.048-18.737)	0.871	0.098	0.036	0.468
	4	5.608(-12.665-23.881)	0.519	0.212	0.114	0.194
Systolic blood pressure	1	-2.695(-22.293-16.902)	0.774	0.005	0.005	0.774
	2	0.692(-20.205-21.588)	0.945	0.07	0.065	0.322
	3	-0.053(-21.72-21.614)	0.996	0.093	0.023	0.563
	4	0.74(-23.579-25.06)	0.949	0.096	0.003	0.851
Diastolic blood pressure	1	-2.092(-15.305-11.12)	0.741	0.007	0.007	0.741
	2	0.766(-13.039-14.572)	0.907	0.109	0.102	0.21
	3	1.637(-12.29-15.564)	0.805	0.177	0.068	0.3
	4	5.194(-9.348-19.736)	0.454	0.29	0.113	0.174

Footnotes: CP= cerebral palsy; MCI=mild cognitive impairment; hs-CRP=high-sensitivity C-reactive protein; BDNF=brain-derived neurotrophic factor; #Semantic fluency (60-second animal naming); WAIS= Wechsler Adult Intelligence Scale, BMI= Body-mass index; bpm= beats per minute; FHS=Framingham heart study; 95% CI=95% confidence interval. * indicates <0.05, ** indicates <0.01, *** indicates <0.001. Model 1: no covariates, Model 2: added gender, Model 3: added years of formal education (in years), Model 4: added chronological age (in years).

Supplementary Table 3D. Associations of natural log-transformed delta FHS score with biomarker, neurocognitive, and anthropometric measures for MCI cohort.

Dependent variable: Biomarkers / Neurocognitive / Anthropometric measures	Models	Independent variable: Natural log-transformed delta FHS score				
		β (95% CI)	*P*-value of regression models	R^2	R^2 Change	*P*-value of R^2 Change
Log-transformed hs-CRP	1	0.073(-0.073-0.218)	0.321	0.019	0.019	0.321
	2	0.069(-0.083-0.22)	0.368	0.019	0.001	0.842
	3	0.07(-0.084-0.223)	0.367	0.02	0.001	0.851
	4	0.071(-0.086-0.227)	0.368	0.02	0	0.91
Log-transformed BDNF	1	0.193(-0.079-0.465)	0.161	0.037	0.037	0.161
	2	0.236(-0.045-0.516)	0.098	0.063	0.026	0.237
	3	0.244(-0.036-0.524)	0.086	0.084	0.021	0.281
	4	0.262(-0.022-0.546)	0.07	0.099	0.015	0.365
Semantic Fluency#	1	-0.795(-1.914-0.324)	0.16	0.037	0.037	0.16
	2	-0.933(-2.093-0.227)	0.113	0.053	0.016	0.356
	3	-0.998(-2.126-0.129)	0.082	0.125	0.073	0.045*
	4	-0.908(-2.043-0.227)	0.115	0.148	0.023	0.251
WAIS-V Block Design	1	0.974(-2.515-4.464)	0.578	0.006	0.006	0.578
	2	-0.367(-3.711-2.978)	0.827	0.164	0.158	0.003**
	3	-0.617(-3.761-2.526)	0.695	0.279	0.114	0.006**
	4	-0.137(-3.187-2.913)	0.929	0.347	0.069	0.026*
BMI (kg/m^2)	1	1.13(-0.251-2.511)	0.107	0.048	0.048	0.107
	2	1.354(-0.069-2.776)	0.062	0.075	0.027	0.225
	3	1.407(-0.008-2.822)	0.051	0.107	0.032	0.184
	4	1.546(0.132-2.96)	0.033*	0.142	0.035	0.158
Resting heart rate (bpm)	1	-1.047(-4.826-2.731)	0.581	0.006	0.006	0.581
	2	-2.086(-5.87-1.698)	0.274	0.087	0.081	0.037*
	3	-2.139(-5.959-1.68)	0.266	0.091	0.004	0.62
	4	-1.833(-5.678-2.012)	0.343	0.115	0.024	0.251
Systolic blood pressure	1	12.413(5.187-19.639)	0.001**	0.183	0.183	0.001**
	2	11.414(3.941-18.886)	0.003**	0.2	0.017	0.3
	3	11.202(3.715-18.69)	0.004**	0.215	0.016	0.319
	4	10.77(3.184-18.356)	0.006**	0.226	0.011	0.41
Diastolic blood pressure	1	-0.11(-4.246-4.026)	0.958	0	0	0.958
	2	-0.722(-4.993-3.549)	0.736	0.024	0.024	0.267
	3	-0.846(-5.123-3.432)	0.693	0.043	0.02	0.309
	4	-0.13(-4.236-3.977)	0.95	0.153	0.109	0.014*

Footnotes: CP= cerebral palsy; MCI=mild cognitive impairment; hs-CRP=high-sensitivity C-reactive protein; BDNF=brain-derived neurotrophic factor; #Semantic fluency (60-second animal naming); WAIS= Wechsler Adult Intelligence Scale, BMI= Body-mass index; bpm= beats per minute; FHS=Framingham heart study; 95% CI=95% confidence interval. * indicates <0.05, ** indicates <0.01, *** indicates <0.001. Model 1: no covariates, Model 2: added gender, Model 3: added years of formal education (in years), Model 4: added chronological age (in years).

Supplementary Table 3E. Associations of BDNF with biomarker, neurocognitive, and anthropometric measures CP cohort.

Dependent variable: Biomarkers / Neurocognitive / Anthropometric measures	Models	Independent variable: Log-transformed BDNF				
		β (95% CI)	*P*-value of regression models	R^2	R^2 Change	*P*-value of R^2 Change
Log-transformed hs-CRP	1	0.146(-0.376-0.668)	0.561	0.022	0.022	0.561
	2	0.143(-0.412-0.697)	0.592	0.022	0	0.95
	3	0.183(-0.412-0.778)	0.52	0.041	0.019	0.606
	4	0.231(-0.429-0.891)	0.464	0.055	0.014	0.668
Semantic Fluency#	1	-2.477(-7.035-2.081)	0.266	0.077	0.077	0.266
	2	-2.57(-7.405-2.264)	0.275	0.079	0.002	0.847
	3	-2.432(-7.667-2.803)	0.336	0.082	0.003	0.842
	4	-1.819(-7.581-3.943)	0.507	0.11	0.029	0.529
WAIS-V Block Design	1	5.312(-10.437-21.062)	0.485	0.031	0.031	0.485
	2	3.078(-12.592-18.748)	0.681	0.15	0.119	0.168
	3	1.436(-15.214-18.087)	0.856	0.183	0.034	0.459
	4	-0.726(-18.985-17.533)	0.933	0.215	0.031	0.484
BMI (kg/m^2)	1	1.097(-1.578-3.772)	0.397	0.045	0.045	0.397
	2	1.297(-1.495-4.09)	0.338	0.078	0.032	0.479
	3	1.325(-1.703-4.352)	0.364	0.078	0	0.945
	4	1.994(-1.199-5.186)	0.2	0.18	0.102	0.225
Resting heart rate (bpm)	1	-10.034(-18.251--1.818)	0.02*	0.295	0.295	0.02*
	2	-11.47(-19.344--3.596)	0.007**	0.426	0.131	0.084
	3	-13.547(-20.933--6.161)	0.001**	0.571	0.144	0.048*
	4	-13.118(-21.346--4.89)	0.004**	0.574	0.003	0.756
Systolic blood pressure	1	6.949(-4.642-18.539)	0.222	0.092	0.092	0.222
	2	5.935(-6.085-17.955)	0.309	0.134	0.042	0.405
	3	5.359(-7.62-18.338)	0.391	0.141	0.007	0.737
	4	6.606(-7.756-20.967)	0.339	0.159	0.018	0.607
Diastolic blood pressure	1	1.361(-6.813-9.536)	0.729	0.008	0.008	0.729
	2	0.306(-7.924-8.536)	0.938	0.108	0.101	0.213
	3	1.551(-6.996-10.097)	0.703	0.182	0.074	0.28
	4	3.719(-5.112-12.549)	0.38	0.302	0.12	0.159
Natural log-transformed delta FHS score	1	0.131(-0.19-0.451)	0.401	0.045	0.045	0.401
	2	0.182(-0.13-0.495)	0.232	0.197	0.152	0.113
	3	0.175(-0.163-0.514)	0.285	0.198	0.001	0.874
	4	0.109(-0.252-0.47)	0.527	0.269	0.071	0.282

Footnotes: CP= cerebral palsy; MCI=mild cognitive impairment; hs-CRP=high-sensitivity C-reactive protein; BDNF=brain-derived neurotrophic factor; #Semantic fluency (60-second animal naming); WAIS= Wechsler Adult Intelligence Scale, BMI= Body-mass index; bpm= beats per minute; FHS=Framingham heart study; 95% CI=95% confidence interval. * indicates <0.05, ** indicates <0.01, *** indicates <0.001. Model 1: no covariates, Model 2: added gender, Model 3: added years of formal education (in years), Model 4: added chronological age (in years).

Supplementary Table 3F. Associations of BDNF with biomarker, neurocognitive, and anthropometric measures MCI cohort.

Dependent variable: Biomarkers / Neurocognitive / Anthropometric measures	Models	Independent variable: Log-transformed BDNF				
		β (95% CI)	*P*-value of regression models	R^2	R^2 Change	*P*-value of R^2 Change
Log-transformed hs-CRP	1	0.07(-0.075-0.214)	0.337	0.017	0.017	0.337
	2	0.074(-0.072-0.221)	0.314	0.023	0.006	0.582
	3	0.074(-0.075-0.223)	0.323	0.023	0	0.997
	4	0.075(-0.077-0.226)	0.326	0.023	0	0.919
Semantic Fluency#	1	-0.928(-2.033-0.177)	0.098	0.051	0.051	0.098
	2	-0.907(-2.028-0.214)	0.111	0.053	0.002	0.72
	3	-0.778(-1.889-0.332)	0.166	0.106	0.053	0.089
	4	-0.854(-1.955-0.247)	0.126	0.146	0.04	0.132
WAIS-V Block Design	1	-2.836(-6.228-0.556)	0.099	0.05	0.05	0.099
	2	-2.321(-5.49-0.848)	0.148	0.197	0.146	0.003**
	3	-1.781(-4.806-1.244)	0.243	0.296	0.099	0.01*
	4	-2.107(-5.001-0.786)	0.15	0.374	0.079	0.016*
BMI (kg/m^2)	1	1.058(-0.32-2.435)	0.129	0.043	0.043	0.129
	2	1.014(-0.38-2.408)	0.15	0.049	0.006	0.555
	3	0.918(-0.489-2.325)	0.196	0.068	0.019	0.311
	4	0.855(-0.559-2.268)	0.23	0.086	0.018	0.325
Resting heart rate (bpm)	1	-2.128(-5.851-1.594)	0.257	0.024	0.024	0.257
	2	-1.778(-5.445-1.89)	0.335	0.082	0.058	0.076
	3	-1.701(-5.436-2.034)	0.365	0.084	0.002	0.76
	4	-1.935(-5.653-1.783)	0.301	0.118	0.034	0.168
Systolic blood pressure	1	3.907(-3.97-11.784)	0.324	0.018	0.018	0.324
	2	4.679(-3.061-12.42)	0.231	0.081	0.063	0.065
	3	5.343(-2.424-13.11)	0.173	0.11	0.029	0.206
	4	5.798(-1.952-13.548)	0.139	0.139	0.029	0.199
Diastolic blood pressure	1	1.694(-2.393-5.781)	0.409	0.013	0.013	0.409
	2	1.948(-2.149-6.046)	0.344	0.038	0.025	0.246
	3	2.266(-1.857-6.39)	0.275	0.063	0.025	0.254
	4	1.821(-2.123-5.765)	0.358	0.167	0.104	0.016
Natural log-transformed delta FHS score	1	0.191(-0.078-0.46)	0.161	0.037	0.037	0.161
	2	0.22(-0.042-0.482)	0.098	0.114	0.078	0.038
	3	0.232(-0.034-0.497)	0.086	0.121	0.007	0.522
	4	0.246(-0.02-0.512)	0.07	0.145	0.024	0.243

Footnotes: CP= cerebral palsy; MCI=mild cognitive impairment; hs-CRP=high-sensitivity C-reactive protein; BDNF=brain-derived neurotrophic factor; #Semantic fluency (60-second animal naming); WAIS= Wechsler Adult Intelligence Scale, BMI= Body-mass index; bpm= beats per minute; FHS=Framingham heart study; 95% CI=95% confidence interval. * indicates <0.05, ** indicates <0.01, *** indicates <0.001. Model 1: no covariates, Model 2: added gender, Model 3: added years of formal education (in years), Model 4: added chronological age (in years).

Supplementary Table 4A. Shared and distinct biomarkers, neurocognitive, and anthropometric measures between adults with CP and MCI.

Dependent variable: Biomarkers / Neurocognitive / Anthropometric measures	Models	Independent variable: CP versus MCI cohorts				
		β (95% CI)	*P*-value of regression models	R^2	R^2 Change	*P*-value of R^2 Change
Log-transformed hs-CRP	1	0.236(0.055-0.418)	0.011*	0.06	0.06	0.011*
	2	0.244(0.058-0.431)	0.011*	0.061	0.001	0.695
	3	0.213(-0.08-0.505)	0.152	0.062	0.001	0.781
Log-transformed BDNF	1	4.222(3.76-4.683)	<0.001***	0.758	0.758	<0.001***
	2	4.247(3.773-4.722)	<0.001***	0.759	0.001	0.62
	3	3.906(3.168-4.645)	<0.001***	0.762	0.003	0.235
Semantic Fluency#	1	-3.848(-7.921-0.225)	0.064	0.032	0.032	0.064
	2	-3.307(-7.471-0.856)	0.118	0.045	0.013	0.236
	3	4.748(-1.447-10.944)	0.132	0.14	0.094	0.001**
WAIS-V Block Design	1	-3.848(-7.921-0.225)	0.064	0.032	0.032	0.064
	2	-3.307(-7.471-0.856)	0.118	0.045	0.013	0.236
	3	4.748(-1.447-10.944)	0.132	0.14	0.094	0.001**
BMI (kg/m^2)	1	-0.3(-2.218-1.619)	0.757	0.001	0.001	0.757
	2	-0.546(-2.508-1.416)	0.582	0.013	0.013	0.253
	3	-1.088(-4.16-1.984)	0.484	0.015	0.002	0.649
Resting heart rate (bpm)	1	-8.21(-12.444--3.977)	<0.001***	0.123	0.123	<0.001***
	2	-8.344(-12.7--3.988)	<0.001***	0.124	0.001	0.779
	3	-4.759(-11.525-2.007)	0.166	0.14	0.016	0.174
Systolic blood pressure	1	16.288(9.633-22.943)	<0.001***	0.183	0.183	<0.001***
	2	17.569(10.817-24.321)	<0.001***	0.206	0.023	0.085
	3	22.19(11.672-32.707)	<0.001***	0.216	0.01	0.259
Diastolic blood pressure	1	-3.18(-6.665-0.305)	0.073	0.03	0.03	0.073
	2	-2.698(-6.259-0.863)	0.136	0.044	0.014	0.217
	3	1.159(-4.335-6.654)	0.676	0.074	0.03	0.073
Natural log-transformed delta FHS score	1	1.787(1.5-2.075)	<0.001***	0.591	0.591	<0.001***
	2	1.868(1.581-2.155)	<0.001***	0.615	0.024	0.012*
	3	2.079(1.632-2.526)	<0.001***	0.621	0.005	0.225

Footnotes: CP= cerebral palsy; MCI=mild cognitive impairment; hs-CRP=high-sensitivity C-reactive protein; BDNF=brain-derived neurotrophic factor; #Semantic fluency (60-second animal naming); WAIS= Wechsler Adult Intelligence Scale; BMI= Body-mass index; bpm= beats per minute; FHS=Framingham heart study; 95% CI=95% confidence interval. * indicates <0.05, ** indicates <0.01, *** indicates <0.001. Model 1: no covariates, Model 2: added gender, Model 3: added years of formal education (in years).

Supplementary Table 4B. Associations of hs-CRP with biomarker, neurocognitive, and anthropometric measures.

Dependent variable: Biomarkers / Neurocognitive / Anthropometric measures	Models	Independent variable: Log-transformed hs-CRP				
		β (95% CI)	*P*-value of regression models	R^2	R^2 Change	*P*-value of R^2 Change
Log-transformed BDNF	1	1.46(0.531-2.388)	0.002**	0.085	0.085	0.002**
	2	1.446(0.527-2.366)	0.002**	0.11	0.026	0.086
	3	0.761(0.074-1.448)	0.03*	0.53	0.419	<0.001***
	4	0.405(-0.084-0.895)	0.104	0.768	0.238	<0.001***
Semantic Fluency#	1	-1.993(-3.704--0.281)	0.023*	0.048	0.048	0.023*
	2	-1.985(-3.703--0.268)	0.024	0.051	0.002	0.611
	3	-1.424(-3.106-0.258)	0.096	0.137	0.086	0.002**
	4	-1.414(-3.122-0.294)	0.104	0.137	0	0.936
WAIS-V Block Design	1	-3.744(-7.965-0.476)	0.081	0.029	0.029	0.081
	2	-3.691(-7.885-0.503)	0.084	0.05	0.022	0.125
	3	-2.363(-6.483-1.757)	0.258	0.131	0.081	0.003**
	4	-2.862(-6.986-1.262)	0.172	0.155	0.024	0.091
BMI (kg/m^2)	1	3.308(1.429-5.187)	0.001**	0.104	0.104	0.001**
	2	3.292(1.413-5.17)	0.001**	0.114	0.01	0.293
	3	3.466(1.544-5.388)	0.001**	0.12	0.007	0.385
	4	3.64(1.704-5.576)	<0.001***	0.134	0.014	0.21
Resting heart rate (bpm)	1	-4.533(-9.127-0.061)	0.053	0.035	0.035	0.053
	2	-4.514(-9.126-0.097)	0.055	0.037	0.002	0.621
	3	-2.903(-7.387-1.582)	0.202	0.137	0.1	0.001**
	4	-2.509(-7.027-2.01)	0.273	0.15	0.013	0.223
Systolic blood pressure	1	6.887(-0.611-14.385)	0.071	0.031	0.031	0.071
	2	6.925(-0.598-14.448)	0.071	0.034	0.003	0.544
	3	4.836(-2.633-12.305)	0.202	0.097	0.063	0.008**
	4	2.824(-4.22-9.868)	0.428	0.221	0.124	<0.001***
Diastolic blood pressure	1	-1.356(-5.007-2.295)	0.463	0.005	0.005	0.463
	2	-1.309(-4.935-2.317)	0.476	0.028	0.023	0.117
	3	-0.466(-4.104-3.172)	0.8	0.073	0.045	0.028*
	4	-0.586(-4.275-3.103)	0.753	0.075	0.002	0.649
Natural log-transformed delta FHS score	1	0.757(0.315-1.199)	0.001**	0.099	0.099	0.001**
	2	0.757(0.313-1.201)	0.001**	0.099	0	0.928
	3	0.503(0.115-0.89)	0.011*	0.349	0.25	<0.001***
	4	0.315(0.021-0.609)	0.036*	0.637	0.288	<0.001***

Footnotes: CP= cerebral palsy; MCI=mild cognitive impairment; hs-CRP=high-sensitivity C-reactive protein; BDNF=brain-derived neurotrophic factor; #Semantic fluency (60-second animal naming); WAIS= Wechsler Adult Intelligence Scale, BMI= Body-mass index; bpm= beats per minute; FHS=Framingham heart study; 95% CI=95% confidence interval. * indicates <0.05, ** indicates <0.01, *** indicates <0.00. Model 1: no covariates, Model 2: added gender, Model 3: added years of formal education (in years), Model 4: added “CP VS MCI cohort”

Supplementary Table 4C. Associations of natural log-transformed delta FHS score with biomarker, neurocognitive, and anthropometric measures for CP cohort.

Dependent variable: Biomarkers / Neurocognitive / Anthropometric measures	Models	Independent variable: Natural log-transformed delta FHS score				
		β (95% CI)	*P*-value of regression models	R^2	R^2 Change	*P*-value of R^2 Change
Log-transformed hs-CRP	1	0.225(-0.238-0.688)	0.334	0.019	0.019	0.334
	2	0.241(-0.243-0.724)	0.322	0.02	0.001	0.799
	3	0.263(-0.232-0.757)	0.291	0.026	0.006	0.594
	4	0.539(-0.009-1.088)	0.054	0.108	0.082	0.043*
Log-transformed BDNF	1	0.006(-0.385-0.398)	0.974	0	0	0.974
	2	0.073(-0.33-0.475)	0.719	0.033	0.033	0.2
	3	0.077(-0.336-0.49)	0.709	0.034	0	0.899
	4	0.18(-0.294-0.655)	0.448	0.05	0.016	0.374
Semantic Fluency#	1	4.531(0.238-8.825)	0.039*	0.082	0.082	0.039*
	2	4.995(0.539-9.451)	0.029*	0.095	0.012	0.415
	3	4.766(0.214-9.318)	0.041*	0.102	0.007	0.549
	4	3.708(-1.527-8.943)	0.161	0.115	0.013	0.409
WAIS-V Block Design	1	-5.343(-15.434-4.749)	0.293	0.022	0.022	0.293
	2	-4.943(-15.48-5.593)	0.35	0.024	0.002	0.766
	3	-6.271(-16.829-4.286)	0.238	0.068	0.044	0.138
	4	-7.605(-19.809-4.6)	0.216	0.072	0.004	0.654
BMI (kg/m^2)	1	1.22(-3.955-6.395)	0.638	0.004	0.004	0.638
	2	1.898(-3.456-7.252)	0.48	0.024	0.02	0.322
	3	1.749(-3.735-7.232)	0.525	0.027	0.002	0.745
	4	1.069(-5.271-7.409)	0.736	0.031	0.004	0.661
Resting heart rate (bpm)	1	0.379(-9.848-10.606)	0.941	0	0	0.941
	2	2.642(-7.736-13.021)	0.611	0.057	0.057	0.091
	3	2.416(-8.219-13.052)	0.65	0.058	0.001	0.8
	4	1.442(-10.865-13.75)	0.815	0.061	0.002	0.746
Systolic blood pressure	1	2.847(-7.406-13.099)	0.58	0.006	0.006	0.58
	2	2.457(-8.249-13.163)	0.647	0.008	0.002	0.775
	3	3.167(-7.742-14.076)	0.562	0.02	0.012	0.439
	4	0.346(-12.177-12.87)	0.956	0.038	0.018	0.358
Diastolic blood pressure	1	6.4(0.916-11.884)	0.023*	0.099	0.099	0.023*
	2	6.264(0.535-11.993)	0.033*	0.1	0.001	0.852
	3	6.162(0.29-12.034)	0.04*	0.1	0.001	0.836
	4	5.2(-1.578-11.978)	0.129	0.107	0.007	0.561

Footnotes: CP= cerebral palsy; MCI=mild cognitive impairment; hs-CRP=high-sensitivity C-reactive protein; BDNF=brain-derived neurotrophic factor; #Semantic fluency (60-second animal naming); WAIS= Wechsler Adult Intelligence Scale, BMI= Body-mass index; bpm= beats per minute; FHS=Framingham heart study; 95% CI=95% confidence interval. * indicates <0.05, ** indicates <0.01, *** indicates <0.001. Model 1: no covariates, Model 2: added gender, Model 3: added years of formal education (in years), Model 4: added chronological age (in years).

Supplementary Table 4D. Associations of natural log-transformed delta FHS score with biomarker, neurocognitive, and anthropometric measures for MCI cohort.

Dependent variable: Biomarkers / Neurocognitive / Anthropometric measures	Models	Independent variable: Natural log-transformed delta FHS score				
		β (95% CI)	*P*-value of regression models	R^2	R^2 Change	*P*-value of R^2 Change
Log-transformed hs-CRP	1	0.073(-0.073-0.218)	0.321	0.019	0.019	0.321
	2	0.069(-0.083-0.22)	0.368	0.019	0.001	0.842
	3	0.07(-0.084-0.223)	0.367	0.02	0.001	0.851
	4	0.071(-0.086-0.227)	0.368	0.02	0	0.91
Log-transformed BDNF	1	0.193(-0.079-0.465)	0.161	0.037	0.037	0.161
	2	0.236(-0.045-0.516)	0.098	0.063	0.026	0.237
	3	0.244(-0.036-0.524)	0.086	0.084	0.021	0.281
	4	0.262(-0.022-0.546)	0.07	0.099	0.015	0.365
Semantic Fluency#	1	-0.795(-1.914-0.324)	0.16	0.037	0.037	0.16
	2	-0.933(-2.093-0.227)	0.113	0.053	0.016	0.356
	3	-0.998(-2.126-0.129)	0.082	0.125	0.073	0.045*
	4	-0.908(-2.043-0.227)	0.115	0.148	0.023	0.251
WAIS-V Block Design	1	0.974(-2.515-4.464)	0.578	0.006	0.006	0.578
	2	-0.367(-3.711-2.978)	0.827	0.164	0.158	0.003**
	3	-0.617(-3.761-2.526)	0.695	0.279	0.114	0.006**
	4	-0.137(-3.187-2.913)	0.929	0.347	0.069	0.026*
BMI (kg/m^2)	1	1.13(-0.251-2.511)	0.107	0.048	0.048	0.107
	2	1.354(-0.069-2.776)	0.062	0.075	0.027	0.225
	3	1.407(-0.008-2.822)	0.051	0.107	0.032	0.184
	4	1.546(0.132-2.96)	0.033*	0.142	0.035	0.158
Resting heart rate (bpm)	1	-1.047(-4.826-2.731)	0.581	0.006	0.006	0.581
	2	-2.086(-5.87-1.698)	0.274	0.087	0.081	0.037*
	3	-2.139(-5.959-1.68)	0.266	0.091	0.004	0.62
	4	-1.833(-5.678-2.012)	0.343	0.115	0.024	0.251
Systolic blood pressure	1	12.413(5.187-19.639)	0.001**	0.183	0.183	0.001**
	2	11.414(3.941-18.886)	0.003**	0.2	0.017	0.3
	3	11.202(3.715-18.69)	0.004**	0.215	0.016	0.319
	4	10.77(3.184-18.356)	0.006**	0.226	0.011	0.41
Diastolic blood pressure	1	-0.11(-4.246-4.026)	0.958	0	0	0.958
	2	-0.722(-4.993-3.549)	0.736	0.024	0.024	0.267
	3	-0.846(-5.123-3.432)	0.693	0.043	0.02	0.309
	4	-0.13(-4.236-3.977)	0.95	0.153	0.109	0.014*

Footnotes: CP= cerebral palsy; MCI=mild cognitive impairment; hs-CRP=high-sensitivity C-reactive protein; BDNF=brain-derived neurotrophic factor; #Semantic fluency (60-second animal naming); WAIS= Wechsler Adult Intelligence Scale, BMI= Body-mass index; bpm= beats per minute; FHS=Framingham heart study; 95% CI=95% confidence interval. * indicates <0.05, ** indicates <0.01, *** indicates <0.001. Model 1: no covariates, Model 2: added gender, Model 3: added years of formal education (in years), Model 4: added chronological age (in years).

Supplementary Table 4E. Associations of BDNF with biomarker, neurocognitive, and anthropometric measures CP cohort.

Dependent variable: Biomarkers / Neurocognitive / Anthropometric measures	Models	Independent variable: Log-transformed BDNF				
		β (95% CI)	*P*-value of regression models	R^2	R^2 Change	*P*-value of R^2 Change
Log-transformed hs-CRP	1	0.363(0.04-0.686)	0.028*	0.093	0.093	0.028*
	2	0.375(0.044-0.706)	0.027*	0.096	0.003	0.694
	3	0.374(0.04-0.708)	0.029*	0.098	0.002	0.724
	4	0.358(0.023-0.693)	0.037*	0.12	0.022	0.28
Semantic Fluency#	1	2.21(-0.978-5.398)	0.17	0.037	0.037	0.17
	2	2.208(-1.064-5.48)	0.181	0.037	0	0.994
	3	2.223(-1.054-5.499)	0.179	0.055	0.018	0.342
	4	2.484(-0.723-5.69)	0.126	0.122	0.067	0.065
WAIS-V Block Design	1	4.446(-2.843-11.735)	0.226	0.029	0.029	0.226
	2	4.216(-3.257-11.689)	0.262	0.032	0.002	0.726
	3	4.263(-3.155-11.681)	0.254	0.066	0.035	0.188
	4	4.235(-3.294-11.763)	0.264	0.066	0	0.932
BMI (kg/m^2)	1	3.198(-0.451-6.846)	0.084	0.058	0.058	0.084
	2	3.013(-0.72-6.746)	0.111	0.065	0.006	0.573
	3	3.021(-0.744-6.787)	0.113	0.069	0.004	0.644
	4	3.162(-0.63-6.954)	0.1	0.083	0.014	0.395
Resting heart rate (bpm)	1	2.851(-4.519-10.22)	0.441	0.012	0.012	0.441
	2	1.864(-5.526-9.254)	0.615	0.057	0.045	0.132
	3	1.876(-5.586-9.338)	0.616	0.059	0.002	0.735
	4	2.056(-5.494-9.605)	0.586	0.065	0.006	0.583
Systolic blood pressure	1	-0.517(-7.971-6.937)	0.89	0	0	0.89
	2	-0.251(-7.89-7.388)	0.948	0.004	0.003	0.691
	3	-0.276(-7.961-7.408)	0.943	0.013	0.01	0.493
	4	0.087(-7.616-7.789)	0.982	0.038	0.024	0.28
Diastolic blood pressure	1	-2.219(-6.359-1.922)	0.287	0.023	0.023	0.287
	2	-2.01(-6.246-2.226)	0.345	0.029	0.006	0.574
	3	-1.999(-6.269-2.271)	0.351	0.035	0.006	0.591
	4	-1.738(-5.98-2.505)	0.414	0.075	0.04	0.16
Natural log-transformed delta FHS score	1	0.003(-0.203-0.209)	0.974	0	0	0.974
	2	0.037(-0.167-0.241)	0.719	0.067	0.067	0.066
	3	0.038(-0.165-0.241)	0.709	0.094	0.026	0.242
	4	0.068(-0.111-0.248)	0.448	0.316	0.222	<0.001***

Footnotes: CP= cerebral palsy; MCI=mild cognitive impairment; hs-CRP=high-sensitivity C-reactive protein; BDNF=brain-derived neurotrophic factor; #Semantic fluency (60-second animal naming); WAIS= Wechsler Adult Intelligence Scale, BMI= Body-mass index; bpm= beats per minute; FHS=Framingham heart study; 95% CI=95% confidence interval. * indicates <0.05, ** indicates <0.01, *** indicates <0.001. Model 1: no covariates, Model 2: added gender, Model 3: added years of formal education (in years), Model 4: added chronological age (in years).

Supplementary Table 4F. Associations of BDNF with biomarker, neurocognitive, and anthropometric measures MCI cohort.

Dependent variable: Biomarkers / Neurocognitive / Anthropometric measures	Models	Independent variable: Log-transformed BDNF				
		β (95% CI)	*P*-value of regression models	R^2	R^2 Change	*P*-value of R^2 Change
Log-transformed hs-CRP	1	0.07(-0.075-0.214)	0.337	0.017	0.017	0.337
	2	0.074(-0.072-0.221)	0.314	0.023	0.006	0.582
	3	0.074(-0.075-0.223)	0.323	0.023	0	0.997
	4	0.075(-0.077-0.226)	0.326	0.023	0	0.919
Semantic Fluency#	1	-0.928(-2.033-0.177)	0.098	0.051	0.051	0.098
	2	-0.907(-2.028-0.214)	0.111	0.053	0.002	0.72
	3	-0.778(-1.889-0.332)	0.166	0.106	0.053	0.089
	4	-0.854(-1.955-0.247)	0.126	0.146	0.04	0.132
WAIS-V Block Design	1	-2.836(-6.228-0.556)	0.099	0.05	0.05	0.099
	2	-2.321(-5.49-0.848)	0.148	0.197	0.146	0.003**
	3	-1.781(-4.806-1.244)	0.243	0.296	0.099	0.01*
	4	-2.107(-5.001-0.786)	0.15	0.374	0.079	0.016*
BMI (kg/m^2)	1	1.058(-0.32-2.435)	0.129	0.043	0.043	0.129
	2	1.014(-0.38-2.408)	0.15	0.049	0.006	0.555
	3	0.918(-0.489-2.325)	0.196	0.068	0.019	0.311
	4	0.855(-0.559-2.268)	0.23	0.086	0.018	0.325
Resting heart rate (bpm)	1	-2.128(-5.851-1.594)	0.257	0.024	0.024	0.257
	2	-1.778(-5.445-1.89)	0.335	0.082	0.058	0.076
	3	-1.701(-5.436-2.034)	0.365	0.084	0.002	0.76
	4	-1.935(-5.653-1.783)	0.301	0.118	0.034	0.168
Systolic blood pressure	1	3.907(-3.97-11.784)	0.324	0.018	0.018	0.324
	2	4.679(-3.061-12.42)	0.231	0.081	0.063	0.065
	3	5.343(-2.424-13.11)	0.173	0.11	0.029	0.206
	4	5.798(-1.952-13.548)	0.139	0.139	0.029	0.199
Diastolic blood pressure	1	1.694(-2.393-5.781)	0.409	0.013	0.013	0.409
	2	1.948(-2.149-6.046)	0.344	0.038	0.025	0.246
	3	2.266(-1.857-6.39)	0.275	0.063	0.025	0.254
	4	1.821(-2.123-5.765)	0.358	0.167	0.104	0.016*
Natural log-transformed delta FHS score	1	0.191(-0.078-0.46)	0.161	0.037	0.037	0.161
	2	0.22(-0.042-0.482)	0.098	0.114	0.078	0.038*
	3	0.232(-0.034-0.497)	0.086	0.121	0.007	0.522
	4	0.246(-0.02-0.512)	0.07	0.145	0.024	0.243

Footnotes: CP= cerebral palsy; MCI=mild cognitive impairment; hs-CRP=high-sensitivity C-reactive protein; BDNF=brain-derived neurotrophic factor; #Semantic fluency (60-second animal naming); WAIS= Wechsler Adult Intelligence Scale, BMI= Body-mass index; bpm= beats per minute; FHS=Framingham heart study; 95% CI=95% confidence interval. * indicates <0.05, ** indicates <0.01, *** indicates <0.001. Model 1: no covariates, Model 2: added gender, Model 3: added years of formal education (in years), Model 4: added chronological age (in years).
